# Uniformly aligned flexible magnetic films from bacterial nanocelluloses for fast actuating optical materials

**DOI:** 10.1038/s41467-022-33615-z

**Published:** 2022-10-03

**Authors:** Xiaofang Zhang, Saewon Kang, Katarina Adstedt, Minkyu Kim, Rui Xiong, Juan Yu, Xinran Chen, Xulin Zhao, Chunhong Ye, Vladimir V. Tsukruk

**Affiliations:** 1 State Key Laboratory of New Textile Materials and Advanced Processing Technologies, Wuhan Textile University, Wuhan, 430200 China; 2 School of Materials Science and Engineering, Georgia Institute of Technology, Atlanta, GA 30332-0245 USA; 3 State Key Laboratory of Polymer Materials Engineering, Polymer Research Institute of Sichuan University, Chengdu, 610065 China; 4 School of Physical Science and Technology, Shanghai Tech University, Shanghai, 201210 China

**Keywords:** Magnetic properties and materials, Self-assembly, Biomaterials

## Abstract

Naturally derived biopolymers have attracted great interest to construct photonic materials with multi-scale ordering, adaptive birefringence, chiral organization, actuation and robustness. Nevertheless, traditional processing commonly results in non-uniform organization across large-scale areas. Here, we report magnetically steerable uniform biophotonic organization of cellulose nanocrystals decorated with superparamagnetic nanoparticles with strong magnetic susceptibility, enabling transformation from helicoidal cholesteric (chiral nematic) to uniaxial nematic phase with near-perfect orientation order parameter of 0.98 across large areas. We demonstrate that magnetically triggered high shearing rate of circular flow exceeds those for conventional evaporation-based assembly by two orders of magnitude. This high rate shearing facilitates unconventional unidirectional orientation of nanocrystals along gradient magnetic field and untwisting helical organization. These translucent magnetic films are flexible, robust, and possess anisotropic birefringence and light scattering combined with relatively high optical transparency reaching 75%. Enhanced mechanical robustness and uniform organization facilitate fast, multimodal, and repeatable actuation in response to magnetic field, humidity variation, and light illumination.

## Abstract (web summary)

Naturally derived biopolymers attracted great interest to construct photonic materials but traditional processing commonly results in non-uniform organization across largescale areas. Here, the authors report a uniform biophotonic organization of cellulose nanocrystals decorated with superparamagnetic nanoparticles enabling transformation from helicoidal cholesteric to uniaxial nematic phase with near-perfect orientation.

## Introduction

Naturally derived biopolymers produced by plants or animals have attracted great interest to construct well-organized photonic organization with long-range multi-scale ordering, anisotropic birefringence, chiral organization, and high robustness with unique capabilities to control the light propagation, excellent mechanical performance, light-weight properties, and potential biocompatibility/biodegradability^1^. Among those, anisotropic nanocellulose materials such as cellulose nanocrystals (CNCs) are an excellent example of structural biopolymers due to a combination of mechanical and optical properties for various photonic applications such as camouflaging, energy harvesting, bio-sensing, optical communication, and actuating^2^. These biomaterials are generally produced by controlled acid hydrolysis of cellulosic biomass that possess one-dimensional (1D) nanostructures of 5–20 nm in width and several hundred nanometers in length^3^. Above a threshold concentration (C^*^), CNCs can self-organize into microscopic domains of liquid crystalline (LC) phase (tactoids) with chiral nematic (cholesteric) order, which coexisted with the isotropic phase^4^. Upon further solvent evaporation, these LC tactoids are subjected to coalescence, nucleation, and eventually fusing into helical ordered domains in eventually dried solid films with frozen random texture of individual tactoids with sharp domain boundaries^5^. The spontaneous random domain morphology generally leads to non-uniformity of chiral nematic phase of CNCs at large scale, excessive light scattering, and compromised optical clarity and actuating ability^6^.

Precise control of the orientation in photonic organization across large-area is critical for the realization of long-range order, uniform optical response for defect-free optical grade materials. Significant progress has been reported to control the orientation of CNCs by applying mechanical shearing^7^, magnetic field^8^, capillary forces^9^, or electrical field^10^. It has been shown that CNCs can be modestly oriented with mechanical shearing^11^. The electric field-triggered alignment of CNCs is usually limited to nonpolar liquid phase in which CNCs are difficult to uniformly disperse^12^. Finally, orientation by capillary forces might result in film cracking during final drying^9^ that requires an additional crosslinking reinforcement.

Specifically, magnetic field-induced alignment offers a feasible technology to control CNC orientation in bulk suspensions and in thin films based on the intrinsic anisotropic diamagnetic susceptibility of CNCs^13^, but this orientation generally requires a very strong magnetic field (ca. 17–28 T), which technically limits scalable processing^14^. Furthermore, with strong magnetic field, CNC tactoids preferentially reorient with the helical axis parallel to the magnetic field, resulting in the modest alignment of chiral nematic phase with low orientational ordering^14^. In the contrast, weak magnetic field (0–1.2 T), cannot align wood pulp or cotton-derived CNCs due to fast Brownian motion of short CNCs^15^. In particular, Kimura et al. reported the orientation of micron-long tunicate-sourced nanofibrils under modest magnetic fields (0.56–1.2 T)^14^. It suggested that anisotropic, needle-like nanocrystals with longer length and larger aspect ratio are able to align under a weak magnetic field^16,17^. Overall, there have been very limited successful attempts to induce CNC orientation using low magnetic field.

As has further been suggested, adding superparamagnetic nanoparticles (MNP), i.e., Fe_3_O_4_ nanoparticles, capable to enhance magnetic susceptibility which is critical in promoting CNC orientation under magnetic field. For instance, Maclachan et al. demonstrated the different magnetic responses of the ordered and disordered phases in lyotropic LC CNC suspensions under a weak magnetic gradient^18^. Berglund et al. fabricated diverse mechanically robust solid and aerogel magnetic materials from cellulose nanofibrils with high ferrite nanoparticle content and unique transport properties^19,20^. Mashkour et al.^21^ prepared unidirectional magnetic paper-like materials from Fe_3_O_4_-coated cellulose fibers by using a permanent magnet. Zhu et al.^22^ showed that the decoration of CNCs with Fe_3_O_4_ nanoparticles significantly reduced the magnetic field strength required to induce the chiral nematic alignment. However, to date, modest orientation order has been achieved and uniformity and optical performance are compromised with agglomeration of magnetic nanoparticles and very high nanoparticle content that makes magnetic cellulose materials black and not optically clear or active.

In this work, we demonstrate that bacterial cellulose nanocrystals (bCNCs) with unusually high aspect ratio of nanocrystals and enhanced magnetic susceptibility due to decoration with Fe_3_O_4_ nanoparticles are capable of assembling into highly oriented mechanically robust and flexible transparent films with anisotropic optical properties. As we observed, the reorganization of bCNC from chiral nematic (cholesteric) ordering with twisted morphology to uniform unidirectional nematic morphology can be realized with doping ratio of Fe_3_O_4_ nanoparticles below 10% due to radial bi-directional flow with extremely high shearing rate and the formation of long chain-like assemblies in magnetic field gradient. Very low doping levels allow the preservation of optical properties such as relatively high transparency, optical birefringence, and inducing uniaxial optical anisotropy and anisotropic light diffraction. In contrast to current examples, near perfect orientation order parameter of CNC, *S* = 0.98, has been achieved under very low magnetic field, below 150 mT. Furthermore, the robust and flexible bCNC-Fe_3_O_4_ films exhibit extremely sensitive and fast response to weak magnetic field, variable humidity, and light illumination due to easily induced anisotropic stresses in near-monodomain thin films.

## Results

### Synthesis and microstructure of bCNCs, Fe_3_O_4_ nanoparticles, and films

**Fig. 1:**
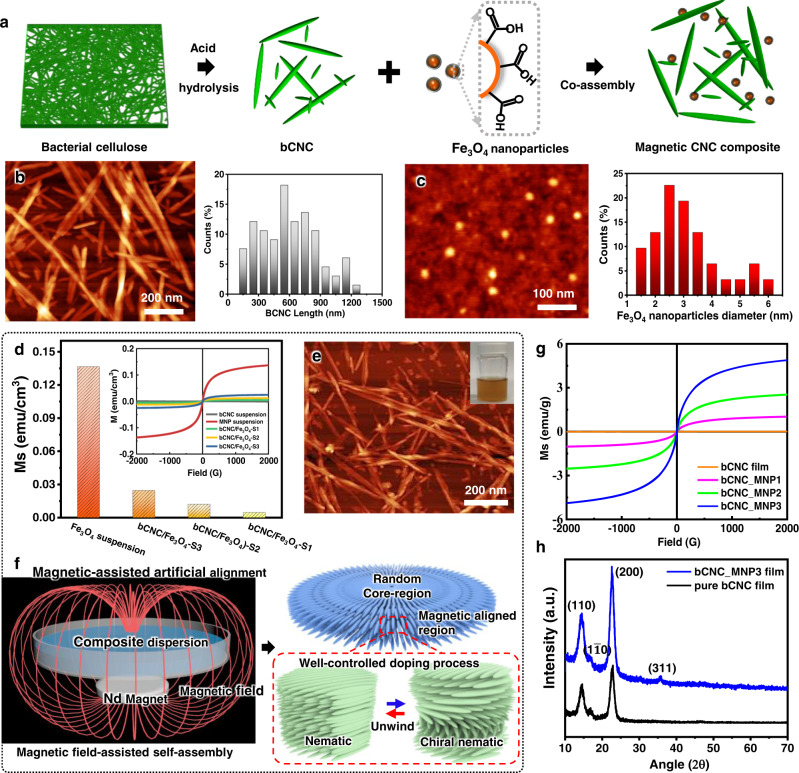
Magnetic-assisted co-assembly of cellulose nanocrystals and magnetic nanoparticles and their properties. **a** Fabrication of magnetic bCNC composites via co-assembly of bCNC and Fe_3_O_4_ nanoparticles; **b** AFM image of as-synthesized bCNCs (left) and their length distribution (right). **c** AFM image of as-synthesized Fe_3_O_4_ nanoparticles (left) and their diameter distribution (right). **d** Ms values of Fe_3_O_4_ suspension and bCNC/Fe_3_O_4_ hybrid suspensions derived from the magnetization curves (inset). **e** AFM image of dispersed bCNC-Fe_3_O_4_ nanostructures. Inset shows the stable aqueous bCNC/Fe_3_O_4_ hybrid suspension. **f** Schematic of magnetic film formation in the presence of a magnetic field (left) and formation of unidirectional nematic ordering that replaces tactoids with chiral nematic organization (right). A disk-like NdFeB magnet is placed beneath Petri dish during drying, the distance is 1.5 cm. **g** Magnetization curves of bCNC film and magnetic bCNC_MNP1-3 films. **h** X-ray data for bCNC film and bCNC-MNP3 film with Miller indices for cellulose nanocrystal ordering, (110) and (200) peaks, along with (311) peak for Fe_3_O_4_ nanoparticles.

Bacterial cellulose was selected as a cellulosic source to obtain high-aspect ratio nanocrystals via acid hydrolysis (Fig. 1a)^23^. Indeed, the resultant bCNCs with 5.4 ± 0.7 nm in diameter possess extremely high aspect ratio (>100) and a broad length distribution from several hundred nanometers to a micron with an average length of 550 ± 170 nm (Fig. 1b, Supplementary Fig. 1). These dimensions largely surpass the traditional wood pulp-derived CNCs with usual length of 100–200 nm and aspect ratio of 20–40^24^. As-synthesized bCNCs show a long-term stability in aqueous suspension against precipitation (inset in Supplementary Fig. 1) due to their highly charged surfaces. It should be pointed out here that, compared with plant-derived short CNCs, much longer bCNCs with larger aspect ratio above 100 has a much lower critical concentration C*^25^. As a result, even initial 0.22 wt% bCNC suspensions can assemble into chiral nematic structures after slow drying and steady increasing concentration (Supplementary Fig. 2), suggesting that LC tactoids are formed with a very low surrounding viscosity.

Based on Kimura’s report^26^, for diamagnetic materials, the magnetic field acquired to overcome the thermal energy and trigger the alignment is inversely proportional to the particle size while proportional to the viscosity of surrounding medium. Therefore, the large size of bCNC contributes to the lower magnetic field required for the alignment. In addition, the longer bCNCs with high aspect ratio increase the anisotropy of diamagnetic susceptibility, further improving the diamagnetic sensitivity of bCNCs with respect to low magnetic field^27^.

The superparamagnetic Fe_3_O_4_ nanoparticles were prepared via the co-precipitation of ferrous (Fe^2+^) and ferric (Fe^3+^) in an aqueous base solution followed by the addition of citric acid to modify their surfaces with the carboxyl groups^28^. Coulombic repulsive interactions between nanoparticles with an average diameter of 3.3 ± 1.2 nm facilitate a fine dispersion and long stability of Fe_3_O_4_ nanoparticles suspensions (Fig. 1c). These Fe_3_O_4_ suspensions exhibit a typical superparamagnetic behavior with extremely small hysteresis loops and coercivity (inset in Fig. 1d). The low magnetization saturation (Ms) of 0.14 emu/cm^3^ is caused by the low concentration of Fe_3_O_4_ nanoparticles (i.e., 0.31 wt%).

Three different magnetic suspensions containing fixed bCNCs content (0.22 wt%) and different Fe_3_O_4_ concentrations were prepared with the Fe_3_O_4_ nanoparticles doping levels being 63, 159, and 318 ppm, respectively. The corresponding hybrid suspensions obtained are denoted as bCNC/Fe_3_O_4_-S1, bCNC/Fe_3_O_4_-S2, and bCNC/Fe_3_O_4_-S3. All bCNC/Fe_3_O_4_ hybrid suspensions show much lower magnetization (0.0048, 0.0123, and 0.0240 emu/cm^3^ for bCNC/Fe_3_O_4_-S1, bCNC/Fe_3_O_4_-S2, and bCNC/Fe_3_O_4_-S3, respectively) (Fig. 1d), due to the low doping level of MNP. AFM image of bCNC-MNP casted from diluted suspension shows that Fe_3_O_4_ nanoparticles are finely co-dispersed with bCNCs (Fig. 1e). Negative charges of both Fe_3_O_4_ nanoparticles with anionic citric acid ligand and bCNCs facilitate long-time stability of the mixed suspension (inset in Fig. 1e)^29^.

Next, drying of the bCNC-Fe_3_O_4_ hybrid suspensions and film formation was conducted under static magnetic field of 150 mT using disk-like Nd-magnet placed beneath Petri dish (Fig. 1f). The visualization of magnetic field with commercial magnetic detection shows the uniform distribution of magnetic field strength with the highest magnetic field concentrated along the circumstance of Nd-magnet (Supplementary Fig. 3a, b). It is worth noting that due to the large size difference in the diameters of Petri dish and NdFeB magnet (4:1 or 60 mm vs 15 mm), there is a magnetic field strength gradient with the peak at the center and gradually decreasing to the Petri dish boundary along the radial direction, i.e., dB/dr (Supplementary Fig. 3c, d). To optimize conditions, we tested different distances between Petri dish and magnet by separating them differently (Supplementary Fig. 4). Too close proximity compromises overall uniformity and results in excessive aggregation and far magnet placement compromises orientation ordering (Supplementary Fig. 4).

After drying in the presence of a magnet (Fig. 1f), we obtained thin magnetic films loaded with 2.8, 6.7, and 12.6 wt% Fe_3_O_4_ nanoparticles, which are denoted as bCNC_MNP1-3 (Supplementary Fig. 5a–c, Supplementary Table 1). All the resultant magnetic films are around 12 μm thick (Supplementary Fig. 6). Decoration of bCNC with magnetic nanoparticles results in a dramatic increase in the magnetization (Fig. 1g). Magnetic strength increases from 0.96 emu/g for bCNC_MNP1 to 4.68 emu/g for bCNC_MNP3 film that is close to those reported in the literature (10–40 emu/g^30^ and slightly lower due to low MNP doping concentration used in this study.

X-ray analysis was further conducted for magnetic bCNC-MNP films to confirm the crystal structure and the presence of nanoparticles (Fig. 1h). The diffuse diffraction peaks reflect the prominent crystalline structure of the traditional CNC materials with primary diffraction peaks at 14.6°, 16.8°, and 22.7°, which correspond to the well-known Miller indexation with the (110), the ($1\bar{1}0$), and the (200) indices^31^. The crystallinity of the bCNC films was calculated using the Segal method^32^:

1 $${CrI}=\frac{({I}_{22.7}-{I}_{18})}{{I}_{22.7}}\times 100\%$$

The crystallinity is very high, 96%, much higher than that for traditional CNCs reported in our previous work (89%) and other reports (75–85%)^31^, indicating high presence of the crystalline phase with fewer defects in much longer bacterial cellulose nanocrystals. Finally, the small diffraction peak at 35.8° corresponds to the (311) reflection for Fe_3_O_4_ crystal lattice and confirms the presence of magnetic nanoparticles in minute quantity^33^.

**Fig. 2:**
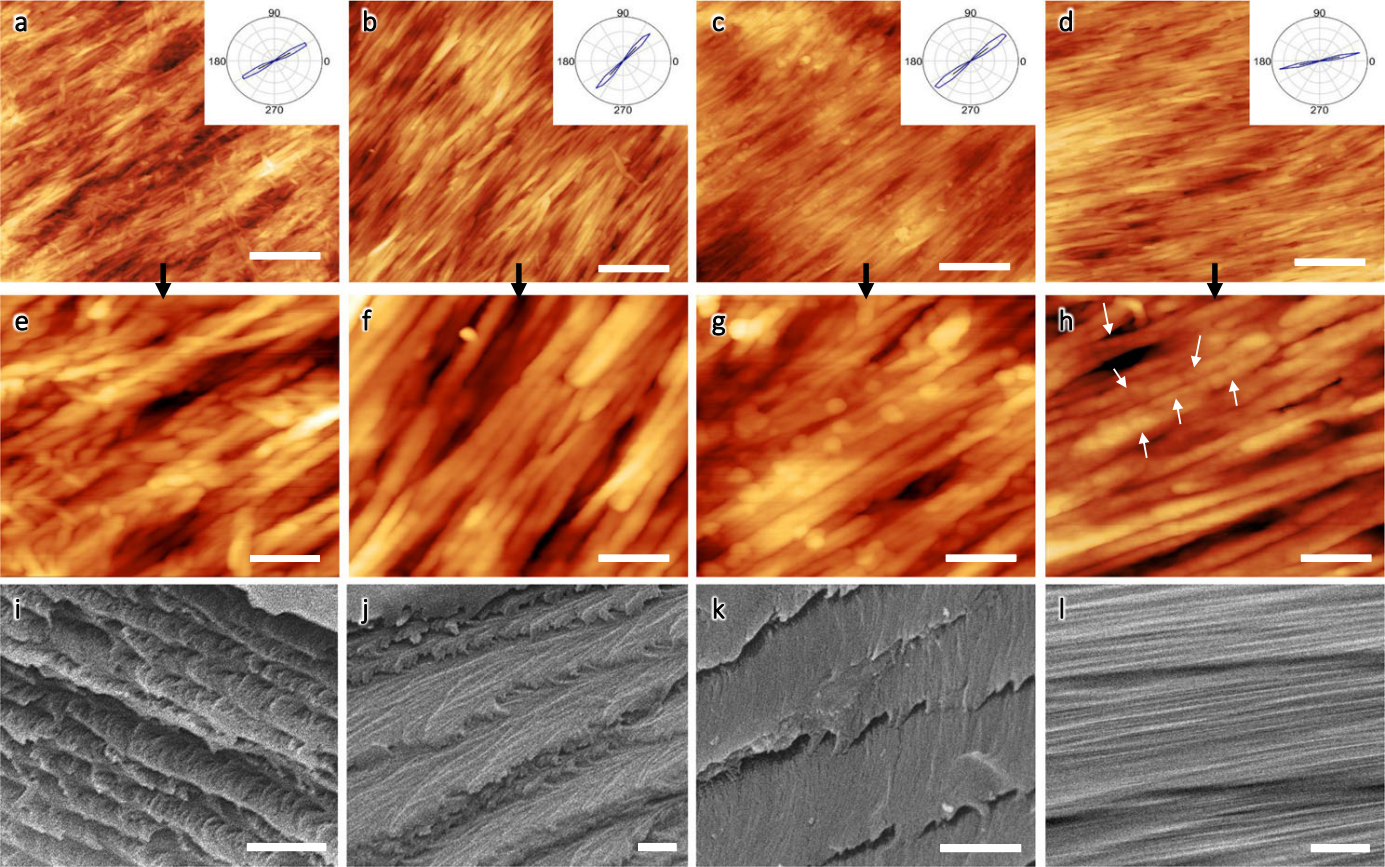
Microstructure transform from chiral nematic to uniaxial nematic organization under magnetic field. AFM topographical images at different magnifications for **a**, **e** bCNC films, **b**, **f** bCNC_MNP1, **c**, **g** bCNC_MNP2, and **d**, **h** bCNC_MNP3 films. Images were collected near the edge of magnet. Scale bars are **a**–**d** 400 nm and **e**–**h** 100 nm, respectively. Insets indicate azimuthal profiles obtained from image analysis of AFM images^37^. Cross-sectional SEM images with characteristic layered morphology of **i** bCNC film, **j** bCNC_MNP1, **k** bCNC_MNP2, and **l** bCNC_MNP3 films. Scale bars are 1 μm.

The surface morphology of all films shows a characteristic uniform texture spanning several micrometers across and nanocrystal bundles at nanoscale (see atomic force microscopy (AFM) images at different scales in Fig. 2a–h, Supplementary Fig. 7a–c). The azimuthal distributions of the nanocrystal orientations, I (Ɵ), show very narrow angular orientation of nanocrystals (insets in Fig. 2a–d, Supplementary Fig. 7d) indicating that the main nanocrystal axes are aligned with high local orientation order^34^. The 2D orientation order parameter, *S*, (Herman’s orientation factor) was derived from this azimuthal distribution as^35,36^:

2 $${S}_{2D}=2\left({{\cos }}^{2}{\theta }_{n}\right)-1$$

As known, orientation order parameter is zero for an isotropic orientation distribution and *S* = 1 for the perfect alignment of the uniaxially oriented fibers^37^. Generally, value of S is within 0.5–0.7 for regular cholesteric and nematic LC phases^38^.

The microscopic surface areas of pure bCNC film show common local orientation texture with narrow azimuthal orientation distribution with high order parameter, *S* > 0.9 and minor defects caused by randomly oriented shorter nanocrystals (Fig. 2a, e). Such high orientation order is in stark contrast to conventional aligned CNC films with modest local orientation of much shorter nanocrystals, showing substantially lower S of ~0.4–0.8 and broader azimuthal distribution. Apparently, high aspect ratio in bacterial nanocellulose crystals (3–5 times higher than that in conventional CNCs) promotes ordered local packing with correlated alignment during transition through LC phase due to sterically-driven energy minimization^39,40^. Moreover, the orientation order further increases after drying in magnetic field with azimuthal distribution becoming very narrow (Fig. 2a–d).

At the highest magnetic nanoparticle loading of 12.6%, the orientation order parameter reaches the highest value of 0.98 with the average orientation deviation angle of about 6° (insets in Fig. 2d), indicating nearly perfect alignment of nanocrystals (theoretical limit of 1.0) never observed for nanocellulose materials. Notably, large-scale images show uniform orientational distribution with few microscopic defects, and no phase separation of magnetic nanoparticles (Supplementary Fig. 7a–c).

Next, high-resolution AFM images of magnetic films reveal the individual and co-assembled Fe_3_O_4_ nanoparticles within CNC bundles (Fig. 2f–h). The characteristic directional texture is preserved in magnetic films with increasing doping concentration of Fe_3_O_4_ nanoparticles (Fig. 2f–h). Higher concentration of magnetic nanoparticles results in dense decoration of nanocrystals and bundles with chain-like magnetic nanoparticles arranged along the nanocrystal main axes (see arrows for individual nanoparticles in Fig. 2h and surface profiles along the long axes of bCNC in Supplementary Fig. 8). We suggest that such chain-like decoration facilitates high magnetic sensitivity and magnetic moment to adapt high orientation under weak magnetic fields with unusually high orientation order parameter.

For fractured films, a characteristic layered Bouligand morphology of chiral nematic organization is observed for pure bCNC film (Fig. 2i, Supplementary Fig. 9a) and bCNC_MNP1 films (Fig. 2j, Supplementary Fig. 9b). Increasing the loading concentration of Fe_3_O_4_ nanoparticles results in disturbed pseudo-layered morphology composed of partially untwisted bundles (Fig. 2k, Supplementary Fig. 9c). The highest concentration of Fe_3_O_4_ nanoparticles in bCNC_MNP3 films promotes uniform densely-packed bCNC fibrillar structures, indicating the formation of uniaxially-oriented nematic organization that replaces characteristic helical organization (Fig. 2l, Supplementary Fig. 9d).

Overall, we suggest that the preferential uniform orientation of MNP decorated bCNCs with high magnetic moment causes unwinding of original helicoidal organization and transition to their uniaxial alignment common for traditional nematic organization (Fig. 1f). Such a chiral nematic-uniaxial nematic reorganization triggered by external magnetic field is a unique feature of high-aspect-ratio magnetic nanocellulose materials studied here. This reorganization is in great contrast to currently reported magnetic cellulose materials with modest orientation of chiral nematic tactoids or fully dark materials with high concentration of ferrite nanoparticles and suppressed structural colors^13^. Magnetic films studied here show a set of optical properties such as semi-transparency, high birefringence, anisotropic light scattering, and suppressed circular polarization as will be discussed below.

### Optical properties of the films: polarization, transmittance, birefringence, and scattering

**Fig. 3:**
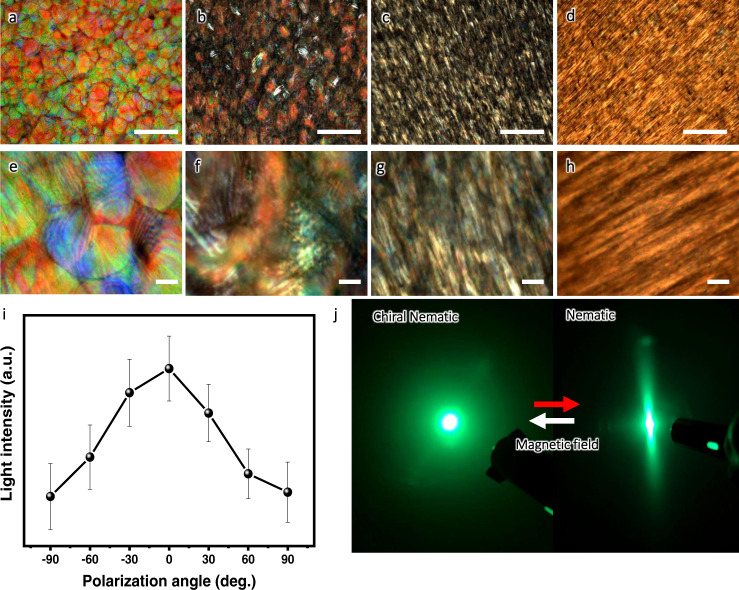
Transformation of optical images and properties under magnetic field. POM images at different magnifications of **a**, **e** bCNC films, **b**, **f** bCNC_MNP1, **c**, **g** bCNC_MNP2, and **d**, **h** bCNC_MNP3 films were taken with crossed polarizers. Scale bars are **a**–**d** 200 μm and **e**–**h** 20 μm, respectively. **i** The azimuthal distribution of polarized light intensity for bCNC_MNP3 film from image 3d. Error bars represent standard deviations, the number of replicates, *n* = 5. **j** The light diffraction of bCNC film (left) and bCNC_MNP3 films (right).

As was pointed above, the resulting magnetic nanocellulose films with distinct low doping of magnetic nanoparticles are clear and uniform with unique optical properties either preserved or transformed. Indeed, polarized optical microscopy (POM) demonstrates that optical birefringence and anisotropy optical textures for all resultant films under crossed polarizers but with different appearances (Fig. 3a–h). In fact, the characteristic random birefringent tactoids with fingerprint-like patterns and sharp boundaries are observed in pure bCNC film (Fig. 3a, e). Similar but more distorted optical texture is observed for magnetic bCNC_MNP1 film with the lowest magnetic nanoparticle content (Fig. 3b, f).

In strike contrast, magnetic films with higher MNP loading exhibit unidirectionally aligned optical texture with highly anisotropic tactoids across large areas (up to 1 mm across) (Fig. 3c, g). Finally, magnetic films with 12.6% of Fe_3_O_4_ nanoparticles possess uniformly aligned texture with high optical anisotropy and without any signs of individual random tactoids (Fig. 3d, h). These uniaxial optical textures with coalesced birefringent domains further confirm the structural reorganization into uniaxial nematic-like organization across macroscopic areas (Fig. 2i).

The azimuthal distribution of transmitted light collected using a hyperspectral microscopy with polarization angle changing from −90° to 90° shows maximum value at 0° indicating unidirectional optical birefringence (Fig. 3i, Supplementary Fig. 10)^41^. The formation of such nematic-type order is similar to the shearing-induced order but is achieved with weak magnetic fields on large areas without directly applying mechanical shearing^11^. Finally, uniaxial organization results in highly anisotropic light diffraction in contrast to isotropic light scattering by original bCNC films with random polydomain tactoids (Fig. 3j, Supplementary Fig. 11).

**Fig. 4:**
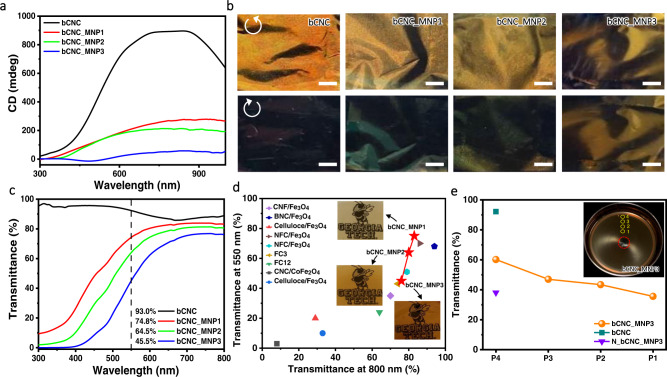
Optical properties of magnetic films. **a** CD spectra of the bCNC films with different loading with magnetic nanoparticles. **b** Photographs of all films viewed under left-handed (top) and right-handed (bottom) circular polarizers. Scale bar: 5 mm. **c** UV–vis spectra of the bCNC films with different magnetic nanoparticle loadings and transmittance data at 550 nm. **d** Comparison in the transmittances at 550 and 800 nm for magnetic films in this study and reported elsewhere^66–71^. Insets show the visibility of patterns under all magnetic films. **e** Transmittance at 550 nm of bCNC_MNP3 film along the radial direction (positions P4–P1 are indicated in inset) and transmittance at the edge of the film (P_4_) in comparison of ordinary cellulose nanocrystal film with and without magnetic nanoparticles.

Next, circular dichroism (CD) of magnetic bacterial nanocellulose films confirms untwisting of initial helical organization and thus supports microscopic morphological studies (Fig. 4a). Indeed, pure bCNC film shows a characteristic strong positive CD signal, indicating the presence of chiral nematic ordering with left-handed helical sense^42^. However, the intensity of CD peak decreases with increasing loading of magnetic nanoparticles and virtually vanishes in bCNC_MNP3 film with the highest Fe_3_O_4_ content (Fig. 4a).

Furthermore, pure bCNC films show brilliant red color under left-handed circular polarizer (LCP) and turn to colorless under right-handed circular polarizer (RCP), demonstrating left-handed circular light polarization across film areas with uniform tactoids (Fig. 4b). However, resultant magnetic films show a diminishing difference in color under both LCP and RCP filters for highest MNP loading content (Fig. 4b), further confirming the formation of uniaxial nematic organization without characteristic circular polarization of chiral nematic phase^43,44^.

On the other hand, we observe that most magnetic particles aggregate at maximum magnetic field region during slow drying (Supplementary Fig. 12). To further verify that the gradient magnetic field causes the redistribution of magnetic nanoparticles, light transmittance in visible spectrum was measured with UV–vis spectroscopy in addition to optical microscopy (Fig. 4c, Supplementary Fig. 12). Direct comparison of UV–vis spectra show that all magnetic films are semi-transparent with light transmittance ranging from 45% to 75% at 550 nm in comparison to 93% for purely bCNC film (Fig. 4c). Specifically, bCNC_MNP1, bCNC_MNP2, and bCNC_MNP3, show the transmittances of 74.8%, 64.5%, and 45.5% at 550 nm, respectively (see inset in Fig. 4c). The gradually-decreased transparency is due to the increased MNP content at similar film thickness. The patterns beneath magnetic films are clearly visible and, overall, all magnetic films studied here possess relatively high optical transparency in comparison to reported magnetic films (Fig. 4d).

Next, in order to investigate the redistribution of the bCNCs-MNP under magnetic field, we measured the UV–vis spectra along the radial direction from central position of the magnet to the edge of the dishes (Supplementary Fig. 13). Notably, the light transmittance at 550 nm decreases gradually from edge to center (from P4 to P1 position) (see plot for bCNC_MNP3 in Fig. 4e and other spectra in Supplementary Fig. 13), indicating gradually increasing concentration of magnetic nanoparticles from edge to center as a result of their movement to the position with the highest magnetic strength. Highly oriented texturing in crossed polarizers is visible in the dry film suggesting the presence of the long-range shearing field caused by flow of magnetic nanoparticles to the central region (Supplementary Fig. 12b, c).

Considering that the central placement of a small magnet beneath the larger Petri dish creates the magnetic field gradient that together with the high nanocrystal magnetization exerts directional magnetic forces, we infer that a magnetically-driven flow along the magnetic field gradient might occur as was, indeed, verified with real-time confocal laser scanning microscopy (CLSM) of labeled suspensions as discussed below.

### Magnetically-driven radial flow and structural reorganization

**Fig. 5:**
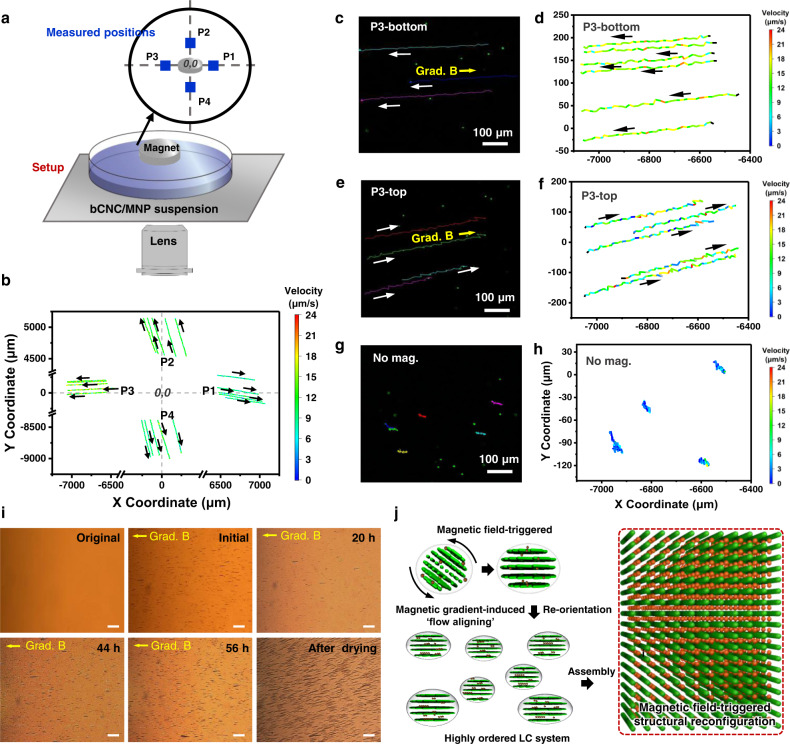
Real-time magnetic field induced flow behavior during the assembly process. **a** Schematic illustration of CLSM measurement setup and captured positions (four blue spots denoted as P1–P4). **b** Trajectories and velocity distributions of the beads located at P1–P4 in the height of 100 μm from the bottom. Tracks and velocities of beads located at P3 with different heights, the bottom layer (**d**), the top layer (**f**), and correspondingly optical images with tracking marks at bottom (**c**) and top (**e**), the white arrow indicated the beads moving directions, the yellow arrow pointed to the magnet. **g**, **h** Beads movements without magnet. The color gradient of the trajectories presented the velocity. The total height of suspension is 1200 μm, bottom layer: 100 μm from the bottom, top layer: 1000 μm from the bottom. **i** Optical microscopy images captured during drying the bCNC-Fe_3_O_4_ hybrid suspension in the presence of magnet. Scale bar: 100 µm. **j** Suggested magnetically-triggered structural reconfiguration to the uniaxial nematic ordering under shearing forces created by circular flow.

In fact, real-time (CLSM) monitoring at different locations (Fig. 5a), upon drying in the presence of magnet, fast flow (linear velocities 10 to 20 μm/s) happens radially outwards from the center (P1–4) (Fig. 5b–f, Supplementary movies 1–4). Drying without magnet resulted in slow near-random local Brownian motion (Fig. 5g, h; Supplementary movie 5). Further measurements captured for one selected position (P3) at different heights of the suspension (bottom and top 100 μm for the total height of 1200 μm) show that the direction of fast flow changed from outward on the bottom to inwards for top layer, closer to the magnet thus cleating circular flow with bottom and top counter flows (Fig. 5c–f, Supplementary movie 3).

We suggest that such unique fast flow circulation is caused by dramatic acceleration of traditional Maragoni flow due to preferential drying the liquid-air-solid contact line along the circumference. This regular evaporation-driven flow is modified and accelerated inwards at the liquid-air interface due to attraction of magnetic cellulose nanocrystals to centrally placed finite magnet. The magnetically-initiated circulation of these fast counter flows at liquid-solid (near bottom) and liquid-air (suspension surface) induces high shearing force and the preferential radial orientation (Fig. 5b), a phenomenon for assembling of CNC suspensions never observed during conventional evaporation-assisted drying.

It is important to note that the effective rate of shearing increases dramatically under the presence of the magnetic field gradient in comparison with that for conventional drying process. Indeed, we measured many-fold higher relative linear velocity of the induced flow under magnetic field (six times) as measure from real-time tracing paths for labeled suspensions with and without magnetic field (Fig. 5c–h).

Moreover, we can estimate diffusion coefficient from the displacement vs time data (Supplementary Fig. 14)^45^. Firstly, as can be concluded from this analysis, the diffusion coefficient calculated for conventional drying in open Petri dish of 7.1 × 10^−7^ cm^2^/s is an order of magnitude higher that common diffusion coefficients measured for sealed bulk suspensions of cellulose nanocrystals (around 1 × 10^−8^ cm^2^/s^46^. Morover, the diffusion coefficient dramatically increases by almost two orders of magnitude (1.1 × 10^−5^ cm^2^/s (for surface flow) and 2.3 × ^−5^ cm^2^/s (for bottom flow) in comparison to drying suspensions without magnetic field gradient (Fig. 5c–h). Such fast and bi-directional field-steerable forced diffusion at opposite interfaces creates high shearing rates that dramatically exceed, by two orders of magnitude, shear rates for conventional slow evaporation-induced drying. Thus, this flow distribution analysis confirms the extremely high shearing rate generated in bCNC-Fe_3_O_4_ hybrid suspension under magnetic field gradient in comparison with conventional “dish casting”.

Finally, the behavior of magnetic suspensions over longer drying period was monitored by optical microscopy in real-time over 3 days of drying under magnetic field (Fig. 5i). Originally, the bCNC-Fe_3_O_4_ hybrid suspension is uniform without any visible nanoparticle aggregation. Placing the magnet initiates the beginning of the formation of chain-like aggregates within very short time (<1 s). At the same time, the aggregates undergo a slow directional drift along the magnetic field gradient with different speeds depending on their size (Supplementary movie 6). At longer time (from 20 hrs to 56 hrs), magnetic nanoparticles start aggregating into much longer (tens of microns) and denser aggregates. Eventually, initial magnetic chains fuse into an unidirectionally aligned very long magnetic aggregates (hundreds of microns) across the entire view field. It is worth to note that such a radial unidirectional organization requires a gradient of magnetic field and suppressed if a larger magnet (comparable size with dish diameter) is used (Supplementary Fig. 15).

In addition, as known, there is a competition between the diamagnetic nature of CNCs and the positive magnetic susceptibility of MNPs under a magnetic field. However, magnetic field gradient and resulting high shearing rate alters the conventional mechanism of tactoid orientation in weak magnetic field to the uniform orientation of nanocrystal bundles not tactoids observed here (Fig. 5j).

Finally, for comparative studies, bCNC_MNP3 films were prepared in the absence of magnetic field (Supplementary Fig. 16). These films show uniform magnetic particle distribution in the film (no radial variation in light transmittance, Supplementary Fig. 13d), traditional Bouligand morphologies (Supplementary Fig. 16a–c) and random orientation of tactoids (Supplementary Fig. 16d, e). Furthermore, optical birefringence texture and positive CD signal confirm preservation of the chiral nematic ordering with left-handed helical sense, which is in sharp contrast with the unidirectional nematic alignment formed in bCNC_MNP3 film fabricated under magnetic field. Moreover, the reorganization to uniaxial nematic organization is not observed in the control films M-bCNC and wCNC_MNP3 fabricated from other types of CNCs with shorter nanocrystals, lower content of MNPs in suspensions, and under larger magnets with uniform magnetic field (Supplementary Table 1, Supplementary Figs. 17–20).

### Structure-reinforced mechanical performance for magnetic films

**Fig. 6:**
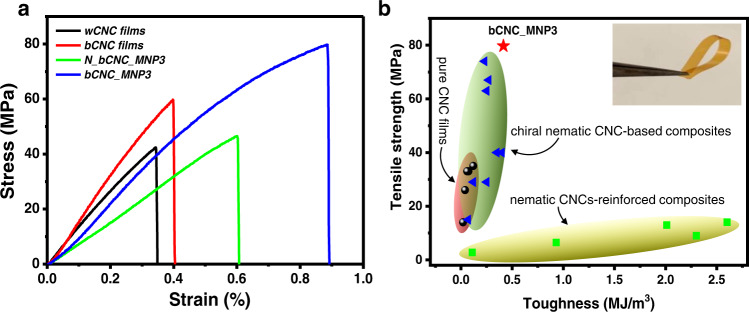
Mechanical performance of reinforced magnetic films. **a** Stress–strain curves of wCNC films, bCNC films, N_bCNC_MNP3 and bCNC_MNP3 composite magnetic films. **b** Ashby plot of tensile strength *vs* toughness of the bCNC_MNP3 composite films and the previously reported CNC-based chiral nematic films, chiral nematic CNC-based composites, and nematic CNCs-reinforced composites. Inset shows folding of the bCNC_MNP3 film.

As known, pristine CNC films show an excessive brittleness and are prone to cracking due to random orientation of tactoids and small nanocrystal overlap lengths that weaken interfacial interactions^2,24^. The rigidity of bCNC causes the brittle fracture of the polydomain films with traditionally small ultimate strain of ca. 0.30% and a modest ultimate strength of ca. 40 MPa, common for CNC chiral nematic materials (Fig. 6a)^3^. In contrast, the uniaxial organization of magnetic films with the uniformly-oriented nanocrystals resulted in much higher mechanical performance with tripled ultimate strain of 0.88 ± 0.04% and doubled ultimate strength of 79.7 ± 4.8 MPa for bCNC_MNP3 (Fig. 6a). As known, mechanical properties of nanocellulose-based materials depend upon relative humidity. Considering that, in this study, all mechanical measurements were conducted at 50 ± 10% relative humidity., Our recent results on humidity dependency of mechanical strength of nanocellulose composites show extreme variation of shearing strength for very dry and wet conditions but stay relative unchanged (within 10–20%) in RH mid-range^47^. Thus, under our experimental conditions, modest variations of mechanical strength is expected that do not affect overall conclusions on many-fold increase in mechanical characteristics.

Strength-toughness Ashby plot combines data from this study and data reported in the literature (Fig. 6b, Supplementary Table 2). Overall, we can conclude that the magnetic films fabricated here possess much higher strength than traditional CNC films^48–52^, CNC-based composites^48–50,52–56^, and CNC-reinforced composites (Fig. 6b)^57,58^. In addition, these magnetic bCNC films are flexible and can be repeatedly folded at 180° without mechanical damages thus opening ways for exploration them as actuating materials as discussed below (inset in Fig. 6b).

### Multi-response actuation of magnetic films

**Fig. 7:**
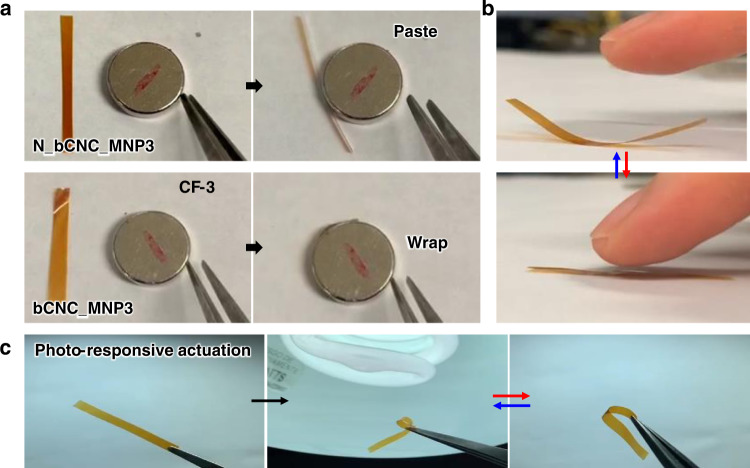
Multi-stimuli responsive behavior of magnetic films. **a** Magnetic responsive bending of bCNC_MNP3 films in the presence of the permanent magnet. **b** Reversible humidity responsive actuation in the presence of a finger. **c** Light-triggered shape transformation in response to local photo-initiated heating.

As known, the magnetic CNC-Fe_3_O_4_ nanocrystals can facilitate added responsive functionalities^59^. Thus, we explored several phenomena for the unidirectional magnetic thin films fabricated in this study. Firstly, flexible and robust thin magnetic films can actuate fast under magnetic field (Fig. 7a). For example, films can spontaneously and quickly wrap around the magnet when placed in the vicinity of a permanent magnet without mechanical damage (Fig. 7a and Supplementary Movie 7). Such quick response and dramatic repeatable shape change with large bending deformation are unique feature of thin flexible magnetic films.

Secondly, the magnetic bCNC_MNP3 films show a quick response to surrounding humidity as demonstrated by fast film actuation in the presence of the finger (Fig. 7b, Supplementary Fig. 21; Supplementary Movie 8). When the finger is approaching, water vapor surrounding the fingertip affects the swelling of the film with one-side preferential swelling as facilitated by the hydrophilic functional groups of nanocrystals and Fe_3_O_4_ nanoparticles which can absorb water molecules (Supplementary Fig. 22). This one-sided interpenetration of water molecules via hydrogen-bonding interactions between bCNCs and Fe_3_O_4_ nanoparticles leads to the asymmetrical film swelling that initiates a fast shape change^60^. Once the finger moved far, the bent film returns to the original shape, indicating very high sensitivity of uniform thin films to the very modest humidity variation caused by the finger's presence (Fig. 7b).

Finally, the photothermal response was observed under light illumination (Fig. 7c; Supplementary Movie 9). Such a fast-triggered reaction can be caused by one-sided light absorption and corresponding thermally-induced bimorph-type stresses due to relatively strong light absorption in the visible wavelength range at near side of the film. In this case, light-induced photothermal stresses can be strong enough to induce shape changes at higher illumination but does not affect the opposite side of the film because of depth-dependent light absorption gradient (Fig. 7c). It is important to note that traditional polydomain CNC films without magnetic nanoparticles and magnetic films composed of different CNC materials show no such response (Supplementary Fig. 23).

## Discussion

In summary, we fabricated unique unidirectionally ordered thin magnetic films from high aspect ratio cellulose nanocrystals decorated with magnetic nanoparticles, which are uniform, robust, flexible, trigger-responsive, and translucent. The mechanisms of magnetic film formation during drying under magnetic field gradient are very different from that reported for conventional evaporation-driven drying processes and drying under uniform magnetic field. We suggest that the unique parallel orientation of magnetically-decorated cellulose nanocrystals in respect to magnetic field direction with untwisted ordering is controlled by extremely high shearing forces and shearing rates of peculiar nature caused by fast radial counter flows under magnetic field gradient (Fig. 5j). High-aspect ratio of nanocrystals further promotes response to shear flow in the initial stage of the tactoid formation during drying and transition across bi-phasic LC state with large “free volume” available for their mobility^61^. The aligned chains of magnetic nanoparticles can further stabilize the nanocrystal nematic ordering with unusually high orientation order parameter^62,63^.

High radial shearing rate is induced by bi-directional radial flow due to the interaction of opposite counter radial-directed flows at liquid–solid bottom and liquid–air top interfaces. High magnetically-triggered shearing rate causes unidirectional nematic orientation of cellulose nanocrystals along radial direction across large areas with parallel orientation in contrast to traditional perpendicular orientation of nanocrystals (equivalent to parallel orientation of tactoids) in uniform magnetic field. Secondary factor, such as significantly increased paramagnetic magnetic moment of decorated nanocrystals further enhance uniaxial organization with near-perfect alignment of nanocrystals with exceptionally high orientation parameter of 0.98, especially considering very weak magnetic field applied, below 150 mT. Such a combination of very high shearing rate and magnetic moment enables assembly of magnetic films with unique transformation from traditional helicoidal organization to uniaxial nematic organization of un-twisted nanocrystals across the cm-sized areas that is rarely observed in magnetically-assisted drying of CNC films.

Furthermore, in contrast to the known carbon-black looking magnetic cellulose materials with high content of magnetic nanoparticles, our thin and flexible magnetic films possess optical qualities such as translucency, high optical birefringence and anisotropic light diffraction. Moreover, they show added multi-functionalities with fast actuation as well as shape morphing under weak magnetic field, local variation of humidity, and photothermally-induced one-side stresses due to anisotropic, one-sided-initiated interfacial mismatch of magnetic, swelling, or thermal origins. We suggest that such robust and flexible magnetic bio-based thin-films with uniform anisotropic organization and associated physical and optical properties present a great potential in the prospective application fields of multi-responsive actuating flexible elements for soft robotics, shape morphing, as soft actuators, and for remote communication and sensing.

## Methods

### Preparation of bacterial cellulose nanocrystals suspensions

The bCNC suspension was prepared by the hydrolysis of dried bacterial cellulose (25 g) with sulfuric acid (64 wt%, 400 mL) at 45 °C for 90 min, followed by diluting ten times with deionized (DI) (18.2 MΩ cm; Synergy UV-R, EMD Millipore) to quench the hydrolysis reaction according to usual procedure^23^. The suspension was settled overnight and the supernatant was decanted. The soluble cellulose residue was washed two times by centrifugation at 3222 × *g* for 8 min to remove the extra sulfuric acid. The crushed-out cellulose was re-dispersed into Nanopure water and then subjected to dialysis (MWCO 14 kDa membrane) against Nanopure water for a week. After that, the suspension was centrifuged at 8952 × *g* for 20 min and the supernatant was collected to obtain purified suspension. The concentration of as-prepared bCNC suspension was calculated to be ca. 0.22 wt%. Before further characterization and co-assembly, the resultant bCNC suspension was subjected to ultrasound treatment for 5 min by tip-sonicator (Qsonica Q125, 50% amplitude).

### Preparation of Fe_3_O_4_ nanoparticles

Fe_3_O_4_ nanoparticles were synthesized via the co-precipitation of ferrous (Fe^2+^) and ferric (Fe^3+^) according to the known procedure^28^. Firstly, FeCl_2_·4H_2_O (0.994 g) and FeCl_3_·6H_2_O (2.703 g) were added into 50 mL of Nanopure water with vigorous stirring for 30 min under nitrogen gas. Subsequently, NH_3_·H_2_O (6 mL) was drop-wise added to the reaction mixture using a syringe. The system was stirred for 30 min. Next, an aqueous solution of citric acid (1.5 g/2.0 mL) was added into the solution with continuous stirring for another 120 min. After reaction, the as-formed Fe_3_O_4_ nanoparticles were washed several times, and separated with external magnetic field to remove the impurities. Finally, the product was collected and re-dispersed in Nanopure water, giving stable suspension with concentration of 0.31 wt%. The Fe_3_O_4_ nanoparticles suspension was subjected to ultrasound treatment for 5 min by tip-sonicator (50% amplitude) before use.

### Films preparation under a weak static magnetic field

bCNC suspensions (0.22 wt%, 10 ml) were mixed with Fe_3_O_4_ nanoparticle suspensions (0.31 wt%) of different volumes (200, 500, and 1000 μL, respectively) to produce a series of mixed suspensions containing identical CNCs content (0.22 wt%) and Fe_3_O_4_ nanoparticles with different concentrations, 63, 129, and 318 ppm in water, respectively. The mixed suspensions were subjected to ultrasound treatment for 5 min, and then drop-casted into a plastic Petri dish (60 × 15 mm) for evaporation-induced self-assembly in the presence of small commercial neodymium (NdFeB) magnet beneath the Petri dish at 1.5 cm distance (diameter 15 mm × thickness 2 mm, with a field strength below 150 mT) (Supplementary Fig. 3). The strength of the magnetic field for the magnet was measured using Gaussmeter (TD 8620, VETUS Industrial Co.) that can measure the flux density of permanent NdFeB magnet with accuracy of ±5%. After drying, obtained bCNC–Fe_3_O_4_ magnetic films were loaded with 2.8, 6.7, and 12.6 wt% Fe_3_O_4_ nanoparticles, which are denoted as bCNC_MNP1, bCNC_MNP2, and bCNC_MNP3, respectively (Supplementary Table 1).

For comparative tests, neat bCNC suspension was drop-cast into a Petri dish above a small commercial neodymium (NdFeB) magnet and bCNC-Fe_3_O_4_ mixture was allowed for evaporating without magnet. In addition, wood pulp-derived CNC (wCNC)-Fe_3_O_4_ mixture was allowed for evaporating with magnet (see different films listed in Supplementary Table 1). After drying, all films were peeled off the Petri dish to obtain free-standing films for further characterization.

### Characterization

AFM images with different resolutions were captured on an ICON AFM microscope (Bruker) with soft tapping mode at 0.7 Hz scan speed^64^. AFM images were acquired with resolution of either 512 × or 1024 × 1024 pixels using AFM probes (MikroMasch, HQ: XSC11/AL BS) with a spring constant of 1.5–2.2 N m^−1^ and a tip radius of ~8 nm. Scans were collected for various surface areas: 5 × 5 µm, 3 × 3 µm, 2 × 2 µm, 1 × 1 µm, and 500 × 500 nm. The suspensions were spin-casted onto a freshly piranha-treated (2:1 ratio of concentrated sulfuric acid and 30% hydrogen peroxide) silicon wafer at 2685 × *g* to obtain dilute nanostructures. The dimensions of bCNCs and Fe_3_O_4_ nanoparticles were determined by using the Gwyddion software. The analysis of orientational order was conducted using GTFiber software, which provides automated AFM image analysis for orientation distribution and *S* value calculation^34^.

The SEM cross sections of fractured films were observed by a Hitachi S-3400N SEM. The specimens were fractured after freezing in liquid nitrogen, and then sputter-coated with a thin layer of gold/palladium.

POM images were collected by an Olympus BX51 microscope in a reflection mode under crossed polarizers. Hyperspectral optical intensity detection system (CytoViva, Inc) equipped with the linear polarizer was used to measure angle-dependent transmitted light intensities of composite films with nematic phase. The CD spectra were collected using an Applied Photophysics Chirascan plus instrument. UV–vis data were obtained on a Shimadzu 3600 UV–NIR spectrophotometer.

The magnetic properties were studied by measuring magnetization as a function of external magnetic field at 300 K using Vibrating sample magnetometer (VSM) (Lakeshore 7404).

XRD analysis was conducted on a PANalytical Empyrean, with a chi-phi-z stage, using a Cu *K*_α_ radiation (*λ* = 1.542 Å), from 10^o^ to 70^o^.

Optical microscope (MicroPublisher 6^TM^, Leica) equipped with 4× objective was employed to monitor the real-time behavior of magnetic suspension in the presence of magnetic field.

CLSM microscope (Olympus FV3000) equipped with 10× objective was utilized to capture the flow movement during the assembly of magnetic suspensions by doping b-CNC: MNPs suspensions with highly fluorescent FITC labeled PS beads with a diameter of 3 μm. The low concentration of the microbeads (below 5 volume%) provides high fluorescent signal without compromising magnetic suspension behavior.

The trace and velocity of beads were quantitatively analyzed by a customized tracking program^65^. During the measurement, the magnet was placed on the top of a Petri dish containing 4 ml suspension (total height of 1200 μm). The real-time video was captured with 488/515 nm of excitation/emission wavelength at a scan rate of 1 frame/s. The time-averaged velocity and trajectory of the beads were tracked and calculated to generate time displacement plots.

Tensile tests (conducted for at least five specimens as 2 × 20 mm strips) were conducted on a Dynamic Mechanical Analyzer (Shimadzu EZ-SX 500) with a speed of 1 mm/min under ambient condition with controlled humidity of 50% at 20 ^o^C. In addition, direct weight measurements show constant film weight with water vapor uptake less than 2%.

## Supplementary information

Supplementary Information

Description of Additional Supplementary Files

Supplementary Movie 1

Supplementary Movie 2

Supplementary Movie 3

Supplementary Movie 4

Supplementary Movie 5

Supplementary Movie 6

Supplementary Movie 7

Supplementary Movie 8

Supplementary Movie 9

## Data Availability

All the data supporting the findings of this study are available within the paper and its Supplementary Information files or from the corresponding author upon request.
