# Supplementary material for: Uniformly aligned flexible magnetic films from bacterial nanocelluloses for fast actuating optical materials
Source: Nat Commun. 2022 Oct 3;13:5804. doi: 10.1038/s41467-022-33615-z (PMC9530119; doi:10.1038/s41467-022-33615-z)
Supplement: Supplementary file 1 — Supplementary Information [file 41467_2022_33615_MOESM1_ESM.pdf]

Supplementary Information

Uniformly Aligned Flexible Magnetic Films from Bacterial Nanocelluloses for Fast Actuating Optical Materials

Xiaofang Zhang,[1,2+] Saewon Kang,[2+] Katarina Adstedt,[2] Minkyu Kim,[2] Rui Xiong,[2,3] Juan Yu,[2] Xinran Chen,[4] Xulin Zhao,[4] Chunhong Ye,[4] Vladimir V. Tsukruk[2]*

1. State Key Laboratory of New Textile Materials and Advanced Processing Technologies, Wuhan Textile University, Wuhan 430200, China

2. School of Materials Science and Engineering, Georgia Institute of Technology, Atlanta, GA 30332-0245, USA

3. State Key Laboratory of Polymer Materials Engineering, Polymer Research Institute of Sichuan University, Chengdu, 610065 China

4. School of Physical Science and Technology, Shanghai Tech University, Shanghai, 201210, China

+ These authors contributed equally: Xiaofang Zhang, Saewon Kang

* Corresponding author: E-mail: vladimir@mse.gatech.edu

Supplementary Table 1. The components and synthesis conditions for all the films studied here. N and Y represent No and Yes, respectively.

Films	Cellulose source	Fe_3O_4 suspension doping (μL)	Magnetic field strength (mT)
bCNC_MNP1	Bacterial cellulose	200	Y
bCNC_MNP2	Bacterial cellulose	500	Y
bCNC_MNP3	Bacterial cellulose	1000	Y
wCNC_MNP3	Wood pulp	1000	Y
N_bCNC_MNP3	Bacterial cellulose	1000	N
bCNC films	Bacterial cellulose	N	N
wCNC films	Wood pulp	N	N
M-bCNC films	Bacterial cellulose	N	Y

Note: bCNC_MNP composite films loaded with 2.8, 6.7, and 12.6 wt% Fe_3O_4 nanoparticles, are denoted as bCNC_MNP1, bCNC_MNP2, and bCNC_MNP3, respectively. M-bCNC films are the pure bCNC films fabricated via self-assembly under weak magnetic field. wCNC_MNP3 films are the woodpulp-sourced CNC/Fe_3O_4 nanoparticle composite films assembled under weak magnetic field. N_bCNC_MNP3 composite films are the bCNC/Fe_3O_4 nanoparticles composite films assembled without applying magnetic field.

Supplementary Table 2. Comparison in tensile strength and toughness between the bCNC-MNP3 in this study and the previously reported chiral nematic CNC films, chiral nematic CNC-based composites, and nematic CNC-reinforced composites.

**Materials**	**Tensile strength (MPa)**	**Toughness (MJ m^{-3})**	**Refs**
CNC film	33	0.074	1
CNC film	33	0.06	2
CNC film	26	0.04	3
CNC film	14	0.02	4
CNC film	35	0.12	5
w-CNC/t-CNC	74	0.23	2
CNC/DMAPS	63	0.25	6
CNC/PVA	67	0.27	7
CNC/latex NPs	29	0.12	1
CNC/Ionic liquid	15	0.07	3
CNC/glycerol	40	0.4	8
CNC/PEG	29	0.25	5
CNC/WPU	42	0.35	9
PVA/CNC25A	6.48	0.93	10
PVA/CNC50A	2.79	0.11	10
PCNC/Glycerol composite dried with magnetic field	9.04	2.3	11
Starch/TCNC/Glycerol composite dried with magnetic field	14.04	2.6	11
Starch/TCNC/PCNC/Glycerol composite dried with magnetic field	13	2.01	11
**bCNC-NMP3**	**79**	**0.415**	**This work**

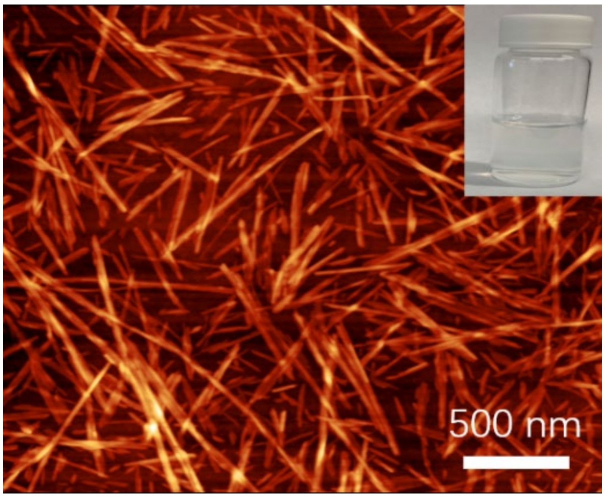

Supplementary Fig. 1. AFM image of pure bCNCs. Inset: stable aqueous suspensions of bCNCs.

Ultralow critical concentration of bCNC suspension

Based on Onsager model,[12] the liquid crystalline (LC) order sets in once the rod concentration reaches a lower critical volume fraction:

$$\emptyset_c^l = 3.3\frac{D}{L}$$

where D is the rod diameter and L is its length. Thus, the greater the aspect ratio (L/D), the smaller the required rod concentration to get a LC order phase.

Prior studies have demonstrated that bCNC with larger aspect ratio exhibited ultralow critical concentration, even more than 1 order of magnitude lower than that of cotton or wood-sourced CNCs.[13] In this work, the resultant bCNCs possess extremely high aspect (>100). The large size of bCNCs enables bCNC suspensions having a very low critical volume fraction.

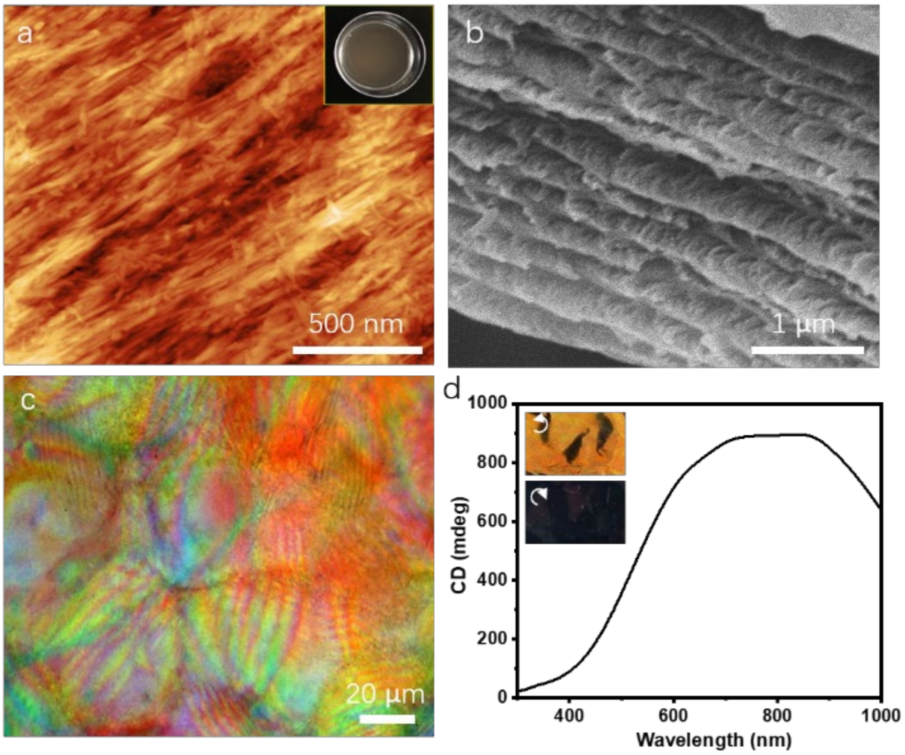

Supplementary Fig. 2. (a) AFM image of bCNC film. Inset is the photography of resultant bCNC film. (b) Cross-sectional SEM image, and (c) POM image of bCNC film. (d) CD spectrum of bCNC film. Inset: the photographs of bCNC film viewed under left-handed (top) and right-handed (bottom) circular polarizers.

Chiral nematic structure formed within pure bCNC suspensions after drying

In this work, drying 0.22 wt% bCNC suspensions resulted in a red-color bCNC film (inset in Supplementary Fig. 2a). The surface AFM image of resultant bCNC film (Supplementary Fig. 2a) shows common local orientation texture. SEM observation reveals the sub-micrometer layered morphology of chiral nematic organization (Supplementary Fig. 2b). POM image exhibits birefringent texture (Supplementary Fig. 2c) and CD spectrum shows positive CD signal (Supplementary Fig. 2d). All the results demonstrate the formation of chiral nematic structure from 0.22 wt% bCNC suspension. It suggests that, even with low initial concentration, bCNCs can assemble into chiral nematic LC phase (i.e., tactoid) instead of are isolated with each other during slow drying.

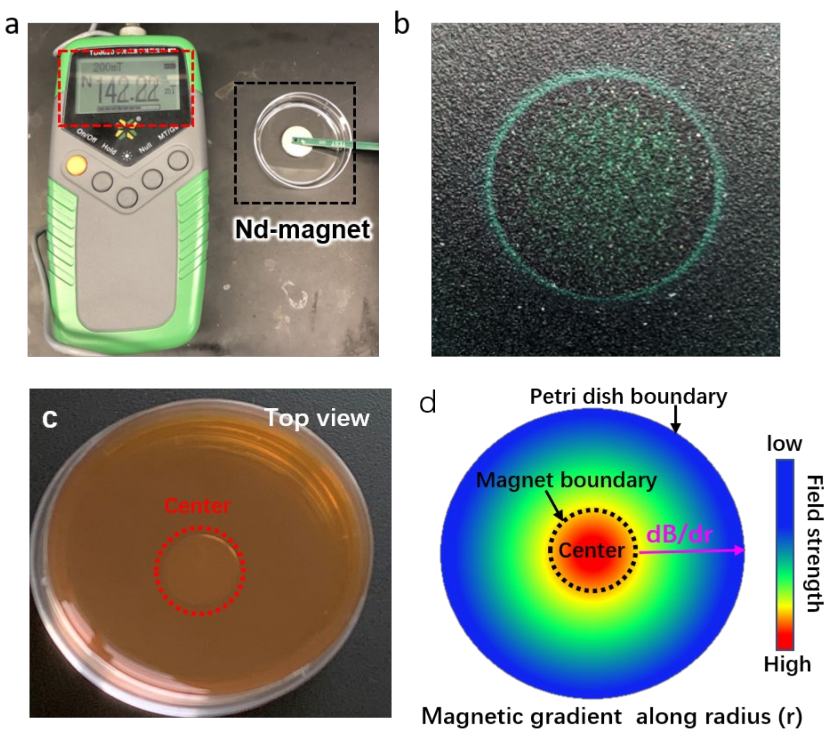

Supplementary Fig. 3. (a, b) Magnetic field strength of < 150 mT was measured for the small NdFeB magnet. (c) Top view of drying bCNC-Fe_3O_4 suspension under static magnetic field a using Nd-magnet placed beneath Petri dish. (d) Schematic diagram of the magnetic gradient along the radius of petri dish.

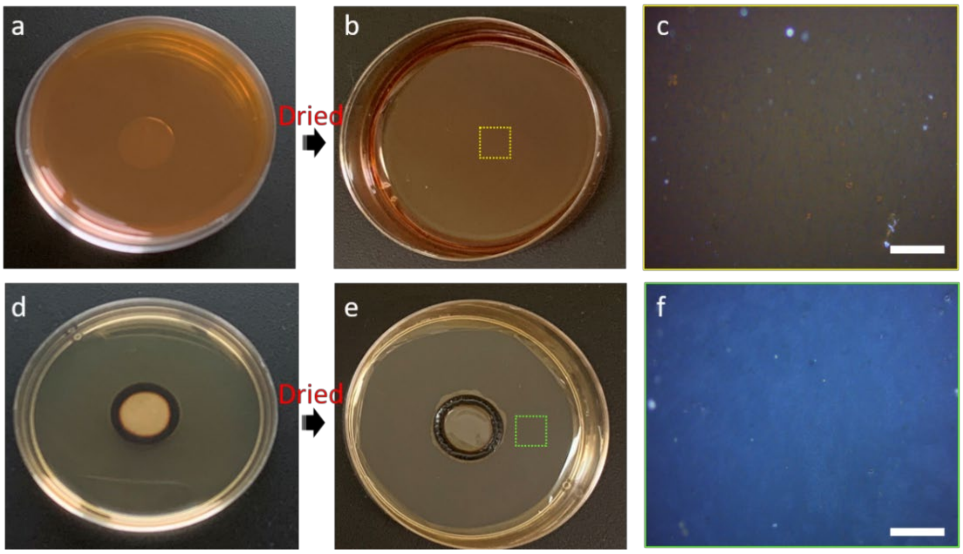

Supplementary Fig. 4. Photographs of drying bCNC-Fe_3O_4 hybrid suspension with Nd-magnet placed beneath Petri dish (a) at distance of 3 cm and (d) in direct contact, without spacing, and (b, e) the corresponding composite films formed and (c, f) POM images. Scale bar: 100 μm.

Drying under different magnetic fields

Real-time observations of magnetically-driven orientational flow are shown in Supplementary Movies 1-6.

Next, we adjusted the distance between Petri dish and Nd-magnet to investigate the influence of Nd-magnet position on the bCNC-MNP composite films formed. Firstly, with the Nd-magnet placed beneath Petri dish with distance of 3 cm (double larger than the 1.5 cm used in the manuscript, Supplementary Fig. 4a), the mixed bCNC-Fe_3O_4 hybrid suspension was dried under a very weak static magnetic field. After drying, a uniform composite film without MNP clumping is formed (Supplementary Fig. 4b). With large distance between petri dish and Nd-magnet, the magnetic strength perceived by bCNC and MNPs was too weak to trigger the radius-directionally flow, as a result, no nematic organization is formed (Supplementary Fig. 4c).

On the contrary, when the Petri dish was put on the Nd-magnet directly (without spacing, Supplementary Fig. 4d), the magnetic strength perceived by bCNC and MNPs increased greatly. In this case, drying mixed bCNC-Fe_3O_4 hybrid suspension resulted in a very un-uniform composite film with obvious phase separation, i.e., almost all the MNPs aggregated along the edge of magnet and no nematic organization formed within bCNC phase (Supplementary Fig. 4f). Based on these results, we concluded that, with too-far or too-close distance between petri dish and Nd-magnet (refers to too weak or too strong magnetic field, respectively), the uni-directional organization cannot be formed.

In this work, the appropriate distance between Nd-magnet and Petri dish (i.e, an appropriate magnetic field strength) combined with the relative high-level doping of MNP, induces an incorporation of negative diamagnetic magnetic force-based alignment of bCNC combining the shearing forces from the MNPs, contributing bCNCs to overcome the twist energy density of the cholesteric phase and transition to the unidirectional alignment.

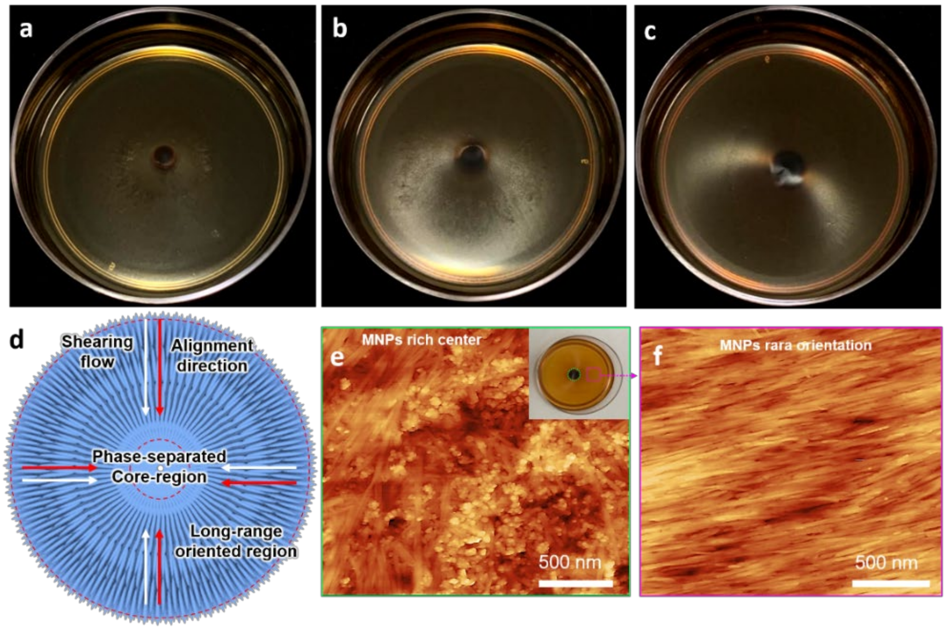

Supplementary Fig. 5. Photographs of as-fabricated (a) bCNC_MNP1, (b) bCNC_MNP2, and (c) bCNC_MNP3 composite films. (d) Schematic illustration of the direction of alignment and magnetic field for long-range ordered phase. AFM images of (e) clumping and (f) uniform region in bCNC-MNP composite films.

Phase separation in bCNC-MNP composite films

As demonstrated before,[14] when the liquid crystalline CNC suspension is doped with MNPs, the isotropic phases can be endowed with significantly higher volume magnetic susceptibility than liquid crystalline (LC) tactoids due to the exclusion effects of tactoids on MNPs. When applying a weak magnetic field gradient, isotropic phases experience unidirectional acceleration along the magnetic field gradient and move to higher-magnetic-field regions. Meanwhile, liquid crystalline tactoids accelerate by magnetic buoyancy forces from the surrounding continuous isotropic phases and move in the opposite direction to lower-magnetic-field regions. Based on this, when the bCNC-MNP hybrid suspension was put in a weak magnetic field gradient, the separation between isotropic phase and liquid crystalline tactoids is accelerated by MNP, that promotes the orientation control of LC tactoids to achieve long-range ordering.

As a result, after drying, the resultant magnetic composite films show the clumping at center and the uniform region out of center are observed (inset in Supplementary Fig. 5a-d). Furthermore, from the AFM image of clumping at center, we can see that MNPs are not bind to bCNCs instead of aggregates with themselves randomly (Supplementary Fig. 5e). In addition, the highly-ordered orientation structure at uniform region is well-maintained without the disturbance from MNPs (Supplementary Fig. 5f). If MNPs are bind to bCNC, then MNPs will homogenously distribute in the whole film, rather than the distinct MNPs phase separation between center and the surrounding. All the results suggest that the affinity between MNP and bCNC is very low due to the strong repulsive force between them.

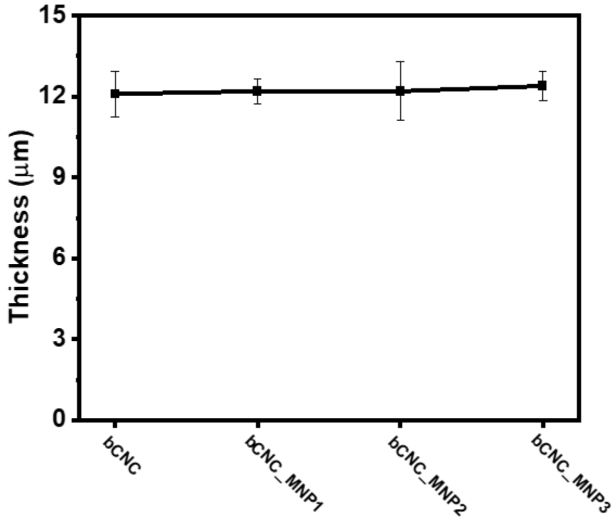

Supplementary Fig. 6. Thickness of resulting bCNC film, and bCNC_MNP1-3 magnetic films. Error bars = standard deviation, the number of replicates, n = 5.

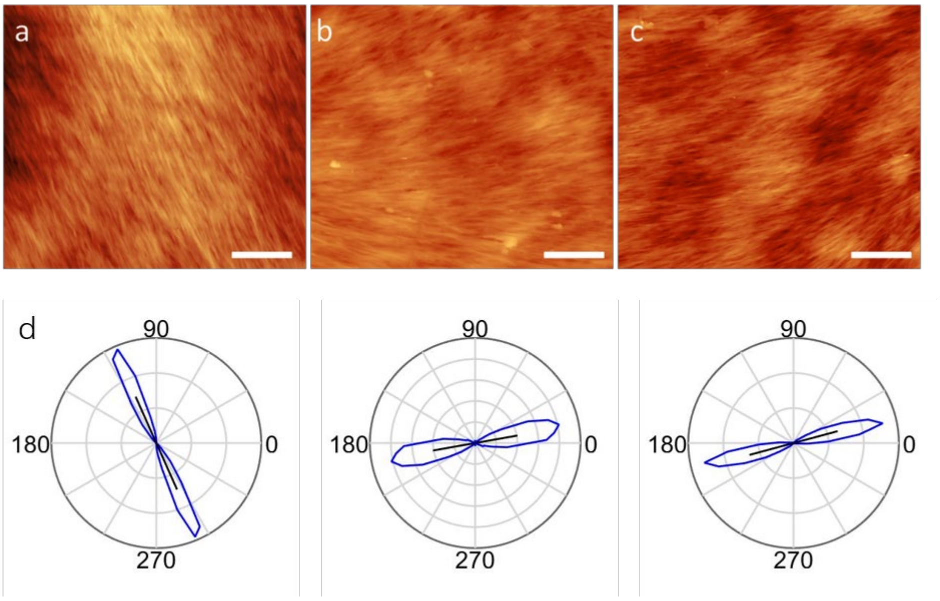

Supplementary Fig. 7. AFM images of the surface morphologies for (a) bCNC_MNP1, (b) bCNC_MNP2, and (c) bCNC_MNP3 composite films. Scale bars are 1 μm. (d) The orientation distribution extracted from the orientation map using image analysis of corresponding AFM images.

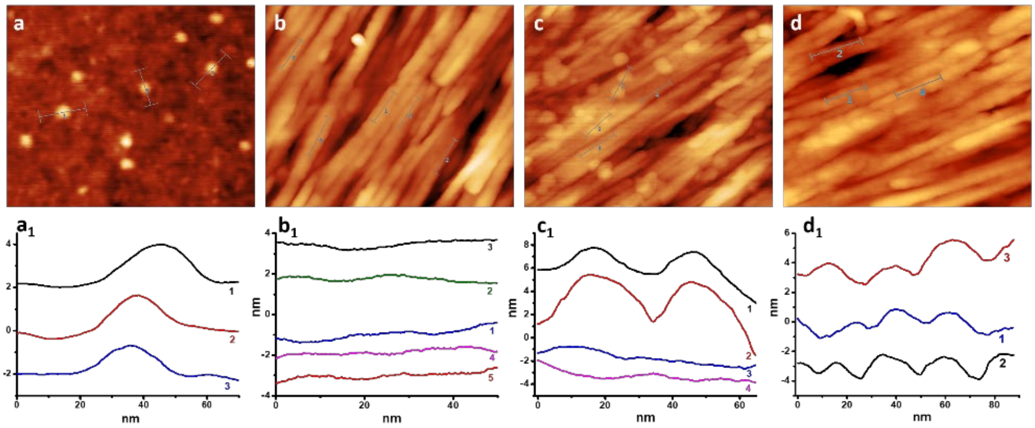

Supplementary Fig. 8. AFM images and cross-sectional/surface profiles for (a, a_1) pure Fe_3O_4 nanoparticles, (b, b_1) bCNC_MNP1, (c, c_1) bCNC_MNP2, and (d, d_1) bCNC_MNP3 composite films.

From the cross-sectional profiles, Fe_3O_4 nanoparticles show an average height variation of ca. 2 nm (a, a_1). Within the straight segments of bCNCs in bCNC_MNP1 composite film, the surfaces of bCNCs are molecularly smooth with linear microroughness along their long axis (b, b_1), suggesting no Fe_3O_4 nanoparticles attached on bCNC surfaces. In bCNC_MNP2 composite film, Fe_3O_4 nanoparticles randomly disperse in the unidirectional organization of bCNCs (c, c_1). Surprisingly, from the surface profiles of bCNCs in bCNC_MNP3 composite film, obviously, Fe_3O_4 nanoparticles are arranged periodically to form chain-like aggregates on bCNC surface effectively with very small spacing and height variation of ca. 2 nm (d, d1) in line with the pristine Fe_3O_4 nanoparticles.

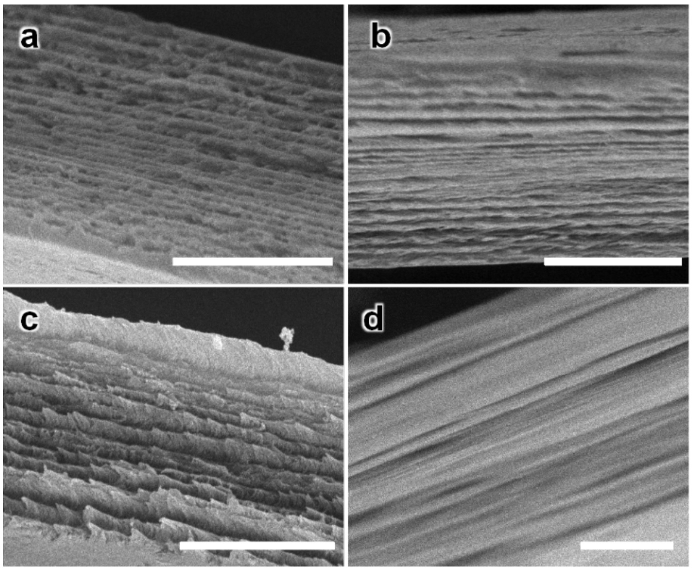

Supplementary Fig. 9. Large-scale cross-sectional SEM images of (a) bCNC films, (b) bCNC_MNP1, (c) bCNC_MNP2, and (d) bCNC_MNP3 composite films. Scale bars are 3 μm.

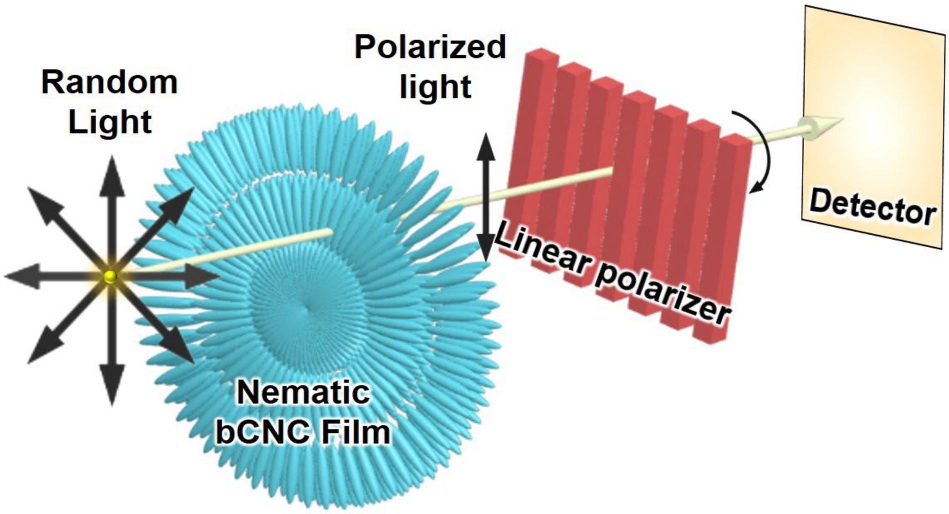

Supplementary Fig. 10. Schematic illustration of the measurement system for polarization angle dependent transmitted light intensity of bCNC_MNP3 film with long-range ordering.

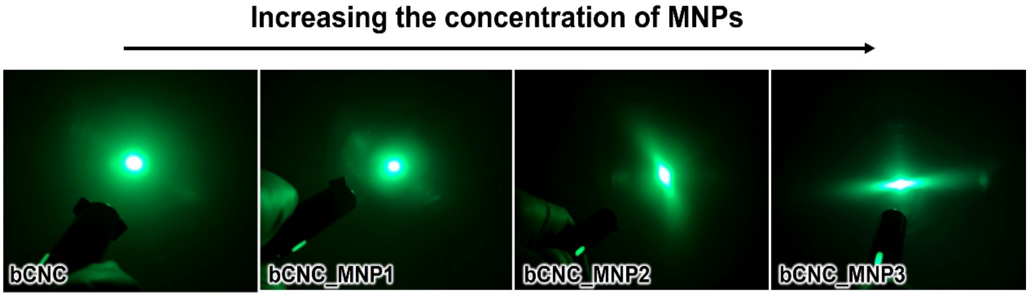

Supplementary Fig. 11. The diffraction pattern of bCNC_MNP composite films fabricated as a function of different MNP concentrations. Pure bCNC films showed circular shaped light scattering while bCNC_MNP3 films exhibited narrow anisotropic pattern in scattered light.

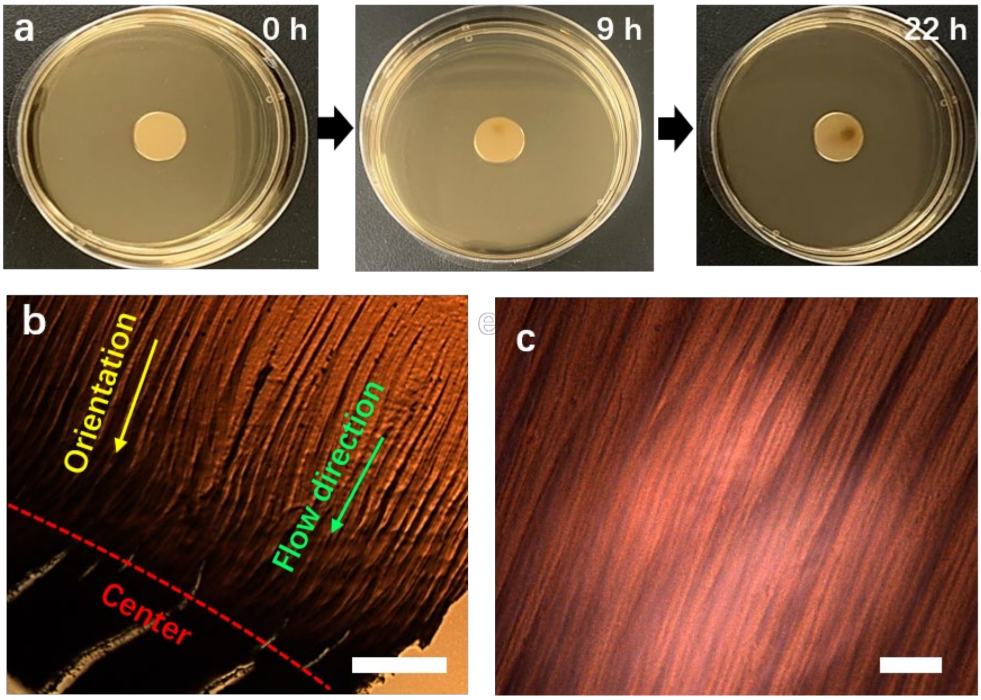

Supplementary Fig. 12. (a) Top view of drying Fe_3O_4 nanoparticle suspension under static magnetic field a using Nd-magnet placed beneath Petri dish. (b, c) POM images of Fe_3O_4 nanoparticles dried under magnetic field. Scale bar: (b) 100 μm and (c) 20 μm.

It is worth noted that due to the large size difference in the diameters of Petri dish and NdFeB magnet, there is a magnetic field strength gradient with the peak at the center and gradually decreasing to the petri dish boundary along the radius, i.e., dB/dr (Supplementary Fig. 3d). Such a magnetic gradient generates a magnetic gradient force,[15] inducing the unidirectional movement of most MNPs along the gradient direction.[14]

To validate this, pure MNP suspension in the petri-dish was dried under a weak magnetic field. During drying, most MNPs slowly move to the high-magnetic field region (i.e, center) (Supplementary Fig. 13a). Due to the polydisperse in size of MNPs and the magnetic gradient, overall, the larger MNPs near the magnet move faster than those smaller ones far away, that produces a velocity and further induces a flow shearing force. The shear, even with slow rate, can trigger "flow aligning" of MNPs to form nematic ordering along the flow direction. Interestingly, POM image of dried MNPs clearly shows a nematic ordering with orientational direction respect to the flow (Supplementary Fig. 13b, c), which is in high consistence with previous report.[16]

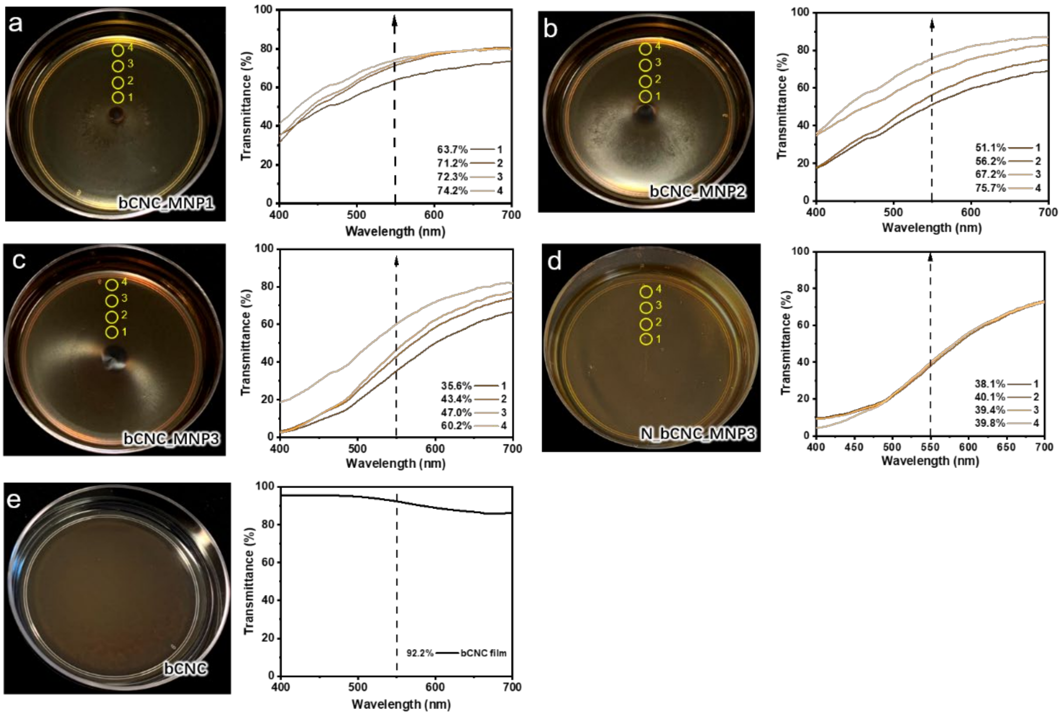

Supplementary Fig. 13. (a-c) Photographs and UV-vis spectra of the bCNC samples with different magnetic nanoparticle loadings dried under magnetic field. (d) Uniform film of bCNC with magnetic nanoparticles dried without magnetic field. (e) bCNC without magnetic nanoparticles,

Summary: bCNC film shows the 92.2% transmittance at 550 nm. After the MNP is incorporated to bCNCs, N_bCNC_MNP3 demonstrates the decreased transmittance of ca. 40% at different positions. For the bCNC_MNP1-3 films, they all display the identical trend in transmittance change depending on the position of the film: transmittance decreases as position of the films move from edge to center (4->1). This result shows that amount of MNP gradually increases as position of the film changes from edge to center.

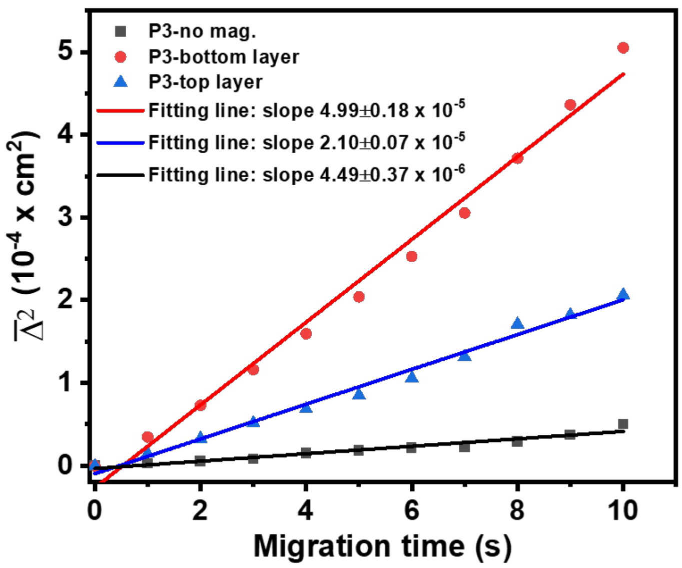

Supplementary Fig. 14. Mean square of the deviation vs diffusion time for the flow: location P3 with different heights in the presence of magnet and without magnet.

Calculation of diffusion coefficient (D) based on Einstein equation for directiional motion:[17]

$$2Dt = \overline{\Delta^2}$$

Where t (s) is the time run and $\overline{\Delta^2}$ (cm^2) is the mean square of the deviation. Based on the Einstein equation, the slope of the linear regression through this data is equal to two times the diffusion coefficient (D). It turns out that the diffusion coefficient of beads under magnetic field is more than an order of magnitude higher than that without magnetic field.

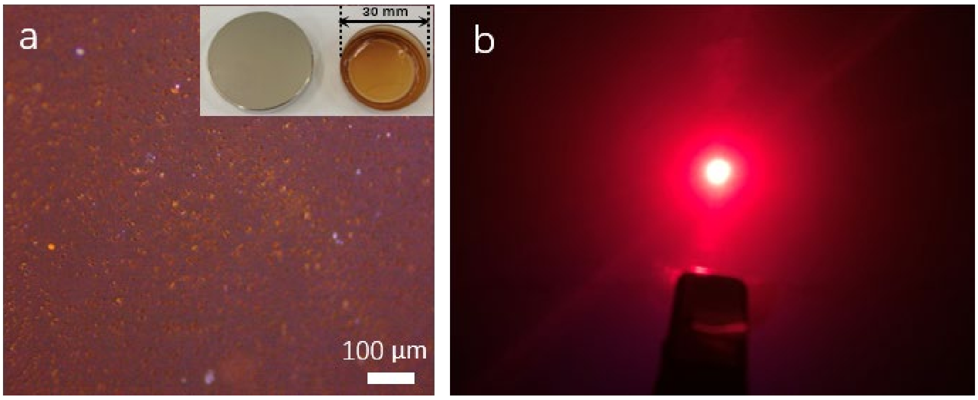

Supplementary Fig. 15. The mixed bCNC-MNP hybrid suspension (bCNC/Fe_3O_4-S3) was drop-casted into a small plastic Petri dish (diameter 30 mm) for evaporation-induced self-assembly in the presence of a big commercial neodymium (NdFeB, diameter 40 mm) magnet beneath the Petri dish: (a) POM image of the resulting film. Inset is the photographs of big magnet used and the resulting film. (b) The diffraction pattern of light reflected by the resulting film.

Influence of radial directional flow on the assembled structures.

In order to investigate the influence of radius-directionally flow on the assemble structure, magnetic hybrid suspension (bCNC/ Fe_3O_4-S3) was drop-casted into a small petri dish (30 mm in diameter) for evaporation-induced self-assembly in the presence of a big magnet (40 mm in diameter) (inset in Supplementary Fig. 15a). Considering the smaller diameter of petri dish compared to the magnet diameter, we speculate that there is no in-of-plane magnetic field strength gradient with the peak at the center and gradually decreasing to the petri dish boundary along the radius. In this case, after drying, the resulting magnetic composite film is uniform without no MNP aggregates at centra (Inset in Supplementary Fig. 15a), suggesting no radius-directionally flow from boundrary to center occured.

Furthermore, POM image shows a random organization structure of the resutling film (Supplementary Fig. 15a), which consequently induces a random diffraction pattern of light (Supplementary Fig. 15b). All the results demonstrate that the highly-ordered nematic organization of bCNC is unachievable in the absence of radius-directionally flow.

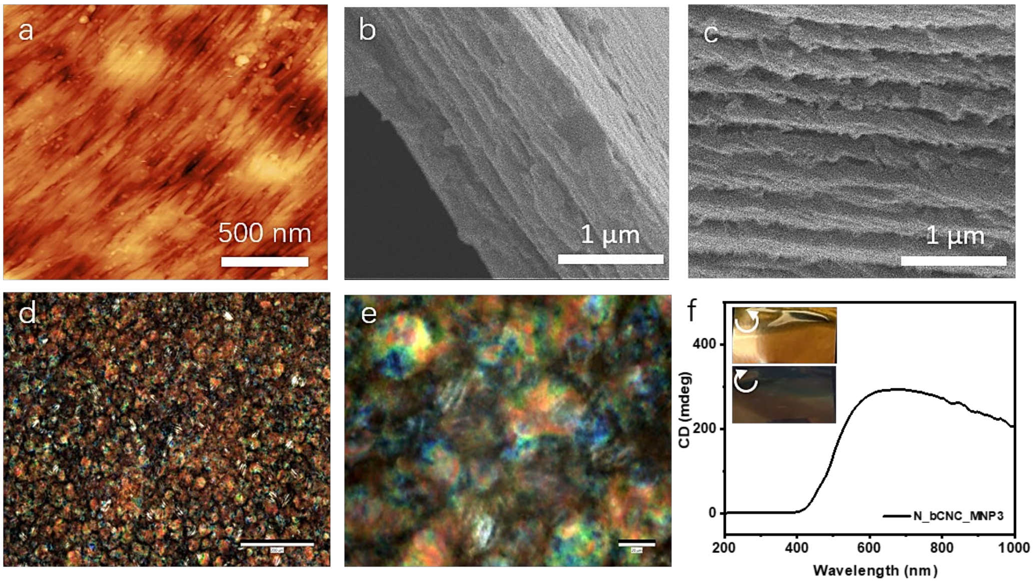

Supplementary Fig. 16. (a) Surface AFM image and (b, c) across-sectional SEM images of N_bCNC_MNP3. (d, e) POM images of N_bCNC_MNP3. Scale bar: (d) 200 μm and (e) 20 μm. (f) CD spectrum of N_bCNC_MNP3, inset is the photographs of N_bCNC_MNP3 films viewed under left-handed (top) and right-handed (bottom) circular polarizers showing difference under left-right circularly polarized lights.

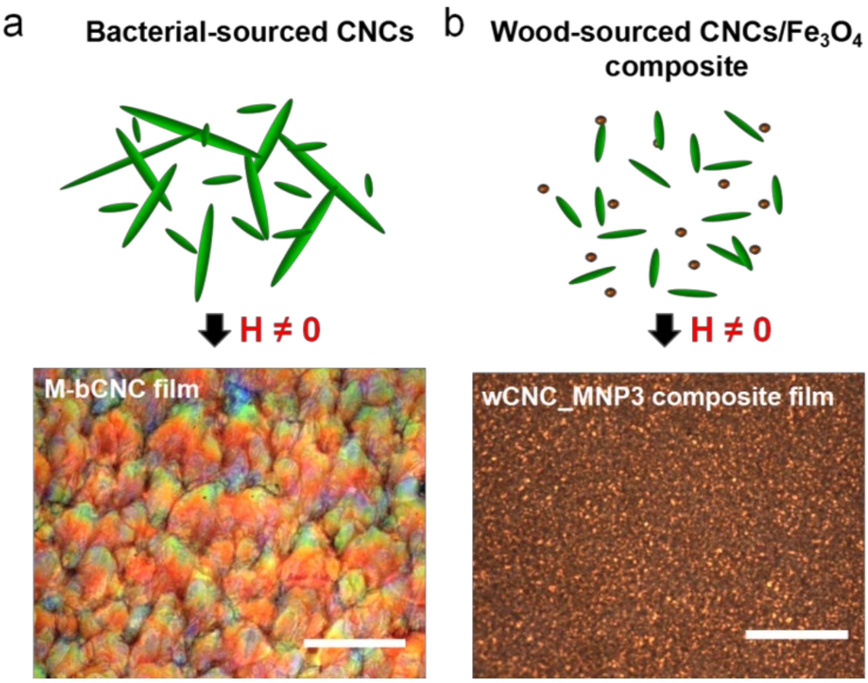

Supplementary Fig. 17. Schematics of the structural reorganizations under different conditions (top) and POM images of (a) M-bCNC films, and (b) wCNC_MNP3. Scale bars are 200 μm.

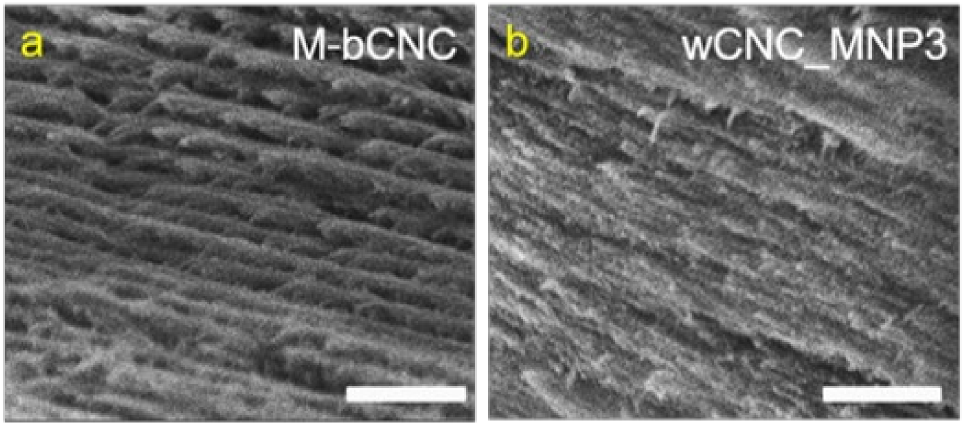

Supplementary Fig. 18. Cross-sectional SEM images of (a) M-bCNC films and (b) wCNC_MNP3. Scale bars are 1 µm.

The role of magnetic field strength and gradient was studied by using a series of magnets (Supplementary Fig. 19, magnet 2 was used thoroughly in this study). In order to investigate the influence of magnetic field strength on the organization structure of magnetic composite films, bCNC-MNP hybrid suspensions (bCNC/Fe_3O_4-S3) were drop-casted in Petri dishes (60 mm in diameter) for evaporation-induced self-assembly in the presence of different magnets beneath the Petri dish at fixed 1.5 cm distance.

Items	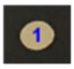	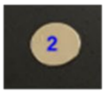	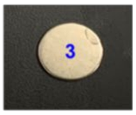	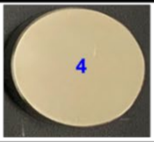
Size (Diameter x Height, (mm x mm))	10 x 1	15 x 2	20 x 4	40 x 10
Magnetic field strength (mT)	~100	~140	~220	~240

Supplementary Fig. 19. The photography, size, and field strength of different commercial magnets. Magnet No. 2 was mostly used in this work.

As we observed, magnetic field strength affects the structure organization of magnetic films (Supplementary Fig. 20). The unidirectionally aligned optical texture is achievable under relative weak magnetic field (Fig 3d, Supplementary Fig. 20d). While it turns into a discontinuous strip-like structure and eventually collapses into the randomly-distributed clusters and short chains with field strength increased and size of the magnet becoming comparable with diameter of the Petri dish making the field gradient vanishing (Supplementary Fig. 20e, f).

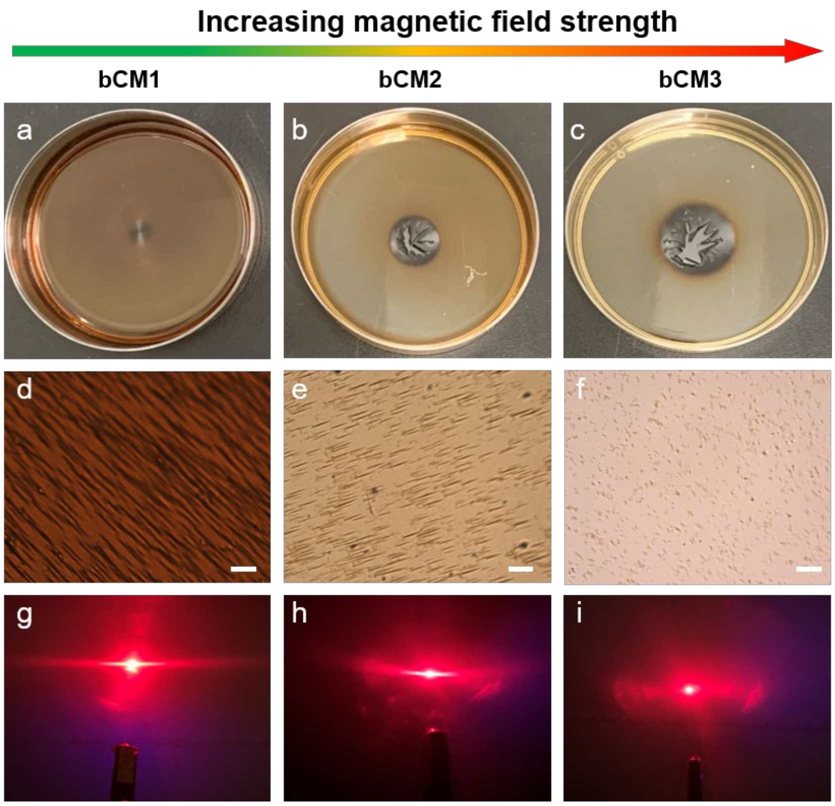

Supplementary Fig. 20. Photographs of (a) bCM1, (b) bCM2 and (c) bCM3 composite magnetic films. POM images of (d) bCM1, (e) bCM2 and (f) bCM3 films. Scale bar: 100 μm. The diffraction pattern of light reflected by the surface of (g) bCM1, (h) bCM2 and (i) bCM3 films.

After drying, the resulting magnetic composite films dired in the presence of magnets No.1, No3, and No.4 are marked as bCM1, bCM2, and bCM3, respectively. Magnet No. 2 is what we used in the work and the corresponding film obtained is bCNC_MNP3. As we can see, with magnetic field increased, bigger aggregate is formed at centra of the resulting films (Supplementary Fig. 20a-c).

Furthermore, POM images show that bCM1 film dried with small magnet No1 exhibits an unidirectionally aligned optical texture identical to that in bCNC_MNP3 (Supplementary Fig. 20d, Fig. 3d). With magnetic field increased (magnet No.3), bCM2 formed exhibits a discontinuous strip-like structure (Supplementary Fig. 20e). As a further increase in magnetic field strength (magnet No.4), MNP clusters and short chains coexist and distribute randomly in the resulting bCM3 film (Supplementary Fig. 20f). Accordingly, with magnetic field increased, the highly-anisotropic light diffraction resulted from the uniaxial organization transforms into the isotropic light scattering reflected by the random organization (Fig. 4g-i).

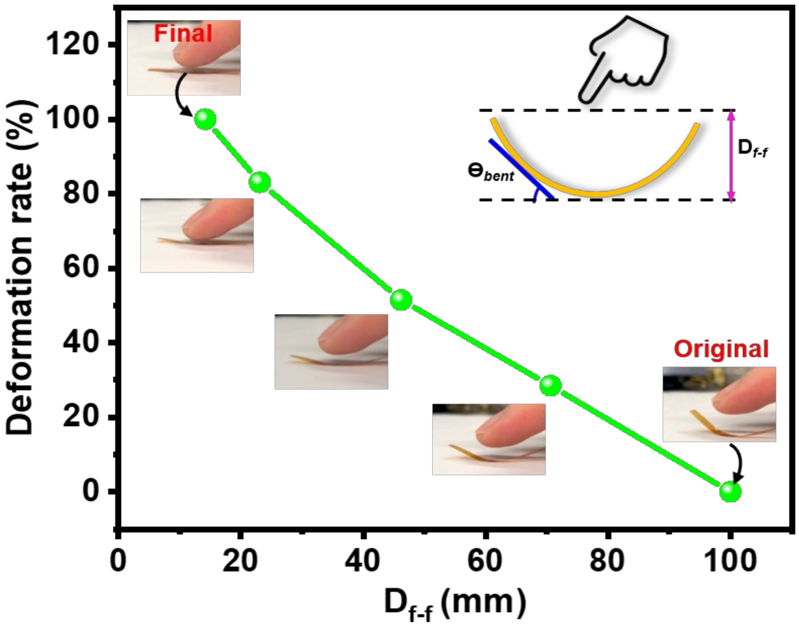

Supplementary Fig. 21. The deforming rate versus D_{f-f} for bCNC_MNP3 composite films.

In this work, the fingertip can drive the bCNC-MNP3 film to deform quickly. The humidity perceived by the film increases with the distance (D) between fingertip and film (D_{f-f}) decreased. Based on this, a curve that depicts the deforming rate versus D_{f-f} has been provided, by which to show the relationship between bending angle of the film and varied humidity qualitatively. The deforming rate is defined as following:

$$Deformation\ rate\ (\%) = \frac{\Delta\theta}{\theta_{original}} \text{x}100\%$$

Where Θ is the bending angle, $\Theta_{original}$ is the initial bending angle before deformation.

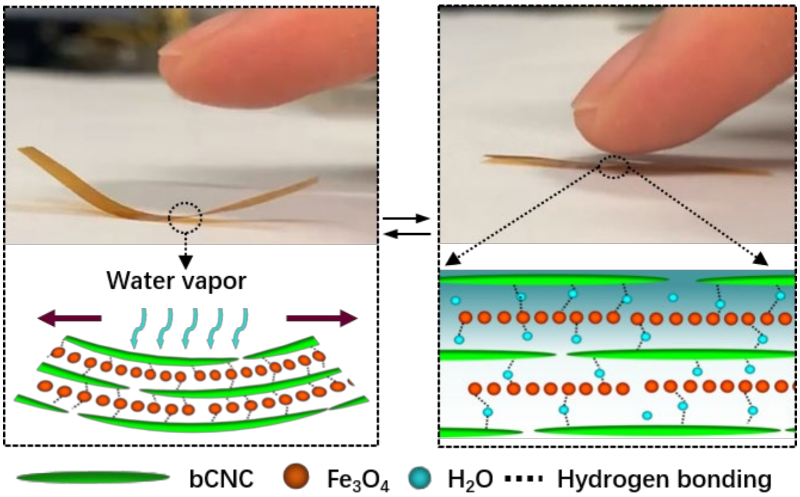

Supplementary Fig. 22. Humidity-induced deforming of magnetic films.

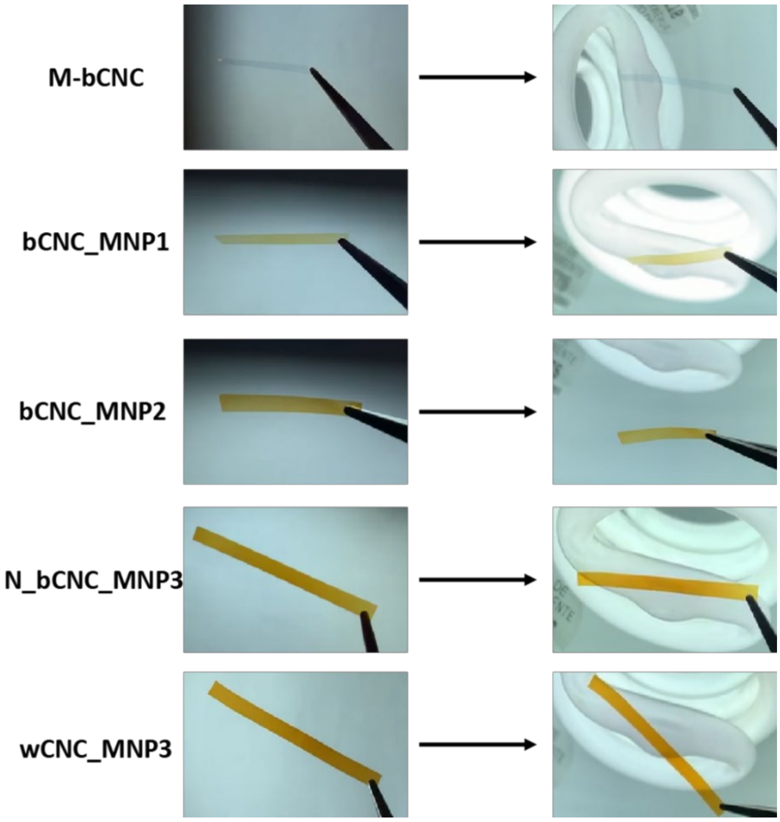

Supplementary Fig. 23. Photographs of photothermal-responsive shape transformation for M-bCNC films, bCNC_MNP1, bCNC_MNP2, N_bCNC_MNP3, and wCNC_MNP3 composite films. These films do not show any shape transformation in response to the light illumination.

Reference

1. Vollick, B., Kuo, P., Thérien-Aubin, H., Yan, N. & Kumacheva, E. Composite cholesteric nanocellulose films with enhanced mechanical properties. *Chem. Mater.* ***29,*** 789-795 (2017).
2. Natarajan, B. et al. Binary cellulose nanocrystal blends for bioinspired damage tolerant photonic films. *Adv. Funct. Mater.* **28**, 1800032 (2018).
3. Liu, P., Guo, X., Nan, F., Duan, Y. & Zhang, J. Modifying mechanical, optical properties and thermal processability of iridescent cellulose nanocrystal films using ionic liquid. *ACS Aappl. Mater. Interfaces* **9**, 3085-3092 (2017).
4. Xiong, R. et al. Flexible, highly transparent and iridescent all-cellulose hybrid nanopaper with enhanced mechanical strength and writable surface. *Carbohydr. Polym.* **113**, 264-271 (2014).
5. Bardet, R., Belgacem, N. & Bras, J. Flexibility and color monitoring of cellulose nanocrystal iridescent solid films using anionic or neutral polymers. *ACS Appl. Mater. Interfaces* **7**, 4010-4018 (2015).
6. Guidetti, G., Atifi, S., Vignolini, S. & Hamad, W. Y. Flexible photonic cellulose nanocrystal films. *Adv. Mater.* ***28***, 10042-10047 (2016).
7. Wang, B. & Walther, A. Self-assembled, iridescent, crustacean-mimetic nanocomposites with tailored periodicity and layered cuticular structure. *ACS nano* **9**, 10637-10646 (2015).
8. Xu, M. et al. Multifunctional chiral nematic cellulose nanocrystals/glycerol structural colored nanocomposites for intelligent responsive films, photonic inks and iridescent coatings. *J. Mater. Chem. C* **6**, 5391-5400 (2018).
9. Wan, H. et al. Rapidly responsive and flexible chiral nematic cellulose nanocrystal composites as multifunctional rewritable photonic papers with eco-friendly inks. *ACS Appl. Mater. Interfaces* **10**, 5918-5925 (2018).
10. Song, W., Liu, D., Nana Prempeh, O. & Song, R. Fiber Alignment and Liquid Crystal Orientation of Cellulose Nanocrystals in the Electrospun Nanofibrous Mats. *Biomacromolecules* **18**, 3273-3279 (2017).
11. Babaei-Ghazvini, A. et al. Effect of magnetic field alignment of cellulose nanocrystals in starch nanocomposites: Physicochemical and mechanical properties. *Carbohydr. Polym.* **247**, 116688 (2020).
12. Onsager, L. The effects of shape on the interaction of colloidal particles. *Ann. N. Y. Acad. Sci.* **51**, 627-659 (1949).
13. Hirai, A., Inui, O., Horii, F. & Tsuji, M. Phase separation behavior in aqueous suspensions of bacterial cellulose nanocrystals prepared by sulfuric acid treatment. *Langmuir* **25**, 497-502 (2009).
14. Wang, P. X., Hamad, W. Y. & MacLachlan, M. J. Liquid crystalline tactoidal microphases in ferrofluids: spatial positioning and orientation by magnetic field gradients. *Chem* **5**, 681-692 (2019).
15. Nguyen, N. T. Micro-magnetofluidics: interactions between magnetism and fluid flow on the microscale. *Microfluid. Nanofluid.***12**, 1-16 (2012).
16. Shrivastav, G. P., Siboni, N. H. & Klapp, S. H. Steady-state rheology and structure of soft hybrid mixtures of liquid crystals and magnetic nanoparticles. *Soft Matter* ***16***, 2516-2527 (2020).
17. P.W. Atkins, Physical Chemistry, 3rd ed., W.H. Freeman and Company, New York, 1986.
