# Supplementary material for: Uniformly aligned flexible magnetic films from bacterial nanocelluloses for fast actuating optical materials
Source: Nat Commun. 2022 Oct 3;13:5804. doi: 10.1038/s41467-022-33615-z (PMC9530119; doi:10.1038/s41467-022-33615-z)
Supplement: Supplementary file 2 — Description of Additional Supplementary Files [file 41467_2022_33615_MOESM2_ESM.pdf]

Title: Supplementary Movie 1
Description: CLSM focused on P1 in the height of 100 μm from the bottom when evaporating hybrid CNC-MNP suspension in the presence of magnet.

Title: Supplementary Movie 2:
Description: CLSM focused on P2 in the height of 100 μm from the bottom when evaporating hybrid CNC-MNP suspension in the presence of magnet.

Title: Supplementary Movie 3:
Description: CLSM focused on P3 in the heights of 100 and 1000 μm from the bottom when evaporating hybrid CNC-MNP suspension in the presence of magnet.

Title: Supplementary Movie 4:
Description: CLSM focused on P4 in the heights of 100 μm from the bottom when evaporating hybrid CNC-MNP suspension in the presence of magnet.

Title: Supplementary Movie 5:
Description: CLSM focused on P3 when evaporating hybrid CNC-MNP suspension without magnet.

Title: Supplementary Movie 6:
Description: Optical microscope monitoring the motion of bCNC-MNPs when evaporating hybrid suspension in the presence of magnet.

Title: Supplementary Movie 7:
Description: Magnetic responsivity of N_bCNC_MNP3 and bCNC_MNP3 when the magnet approaches to the film. bCNC_MNP3 was intimately attached to the magnet with curvature while N_bCNC_MNP3 was simply stuck to the magnet.

Title: Supplementary Movie 8:
Description: Remote stimuli-triggered mechanical actuation of bCNC_MNP3.

Title: Supplementary Movie 9:
Description: Photo-responsive mechanical actuation of bCNC_MNP3.
